# Head and neck cancer treatment outcome priorities: A multi-perspective concept mapping study

**DOI:** 10.1371/journal.pone.0294712

**Published:** 2023-11-30

**Authors:** Waad R. Alolayan, Jana M. Rieger, Minn N. Yoon

**Affiliations:** 1 Faculty of Rehabilitation Medicine, University of Alberta, Edmonton, Alberta, Canada; 2 Institute for Reconstructive Sciences in Medicine (iRSM), Misericordia Community Hospital, Edmonton, Alberta, Canada; 3 Faculty of Medicine and Dentistry, School of Dentistry, University of Alberta, Edmonton, Alberta, Canada; University of Alabama at Birmingham, UNITED STATES

## Abstract

With the increasing focus on patient-centred care, this study sought to understand priorities considered by patients and healthcare providers from their experience with head and neck cancer treatment, and to compare how patients’ priorities compare to healthcare providers’ priorities. Group concept mapping was used to actively identify priorities from participants (patients and healthcare providers) in two phases. In phase one, participants brainstormed statements reflecting considerations related to their experience with head and neck cancer treatment. In phase two, statements were sorted based on their similarity in theme and rated in terms of their priority. Multidimensional scaling and cluster analysis were performed to produce multidimensional maps to visualize the findings. Two-hundred fifty statements were generated by participants in the brainstorming phase, finalized to 94 statements that were included in phase two. From the sorting activity, a two-dimensional map with stress value of 0.2213 was generated, and eight clusters were created to encompass all statements. Timely care, education, and person-centred care were the highest rated priorities for patients and healthcare providers. Overall, there was a strong correlation between patient and healthcare providers’ ratings (r = 0.80). Our findings support the complexity of the treatment planning process in head and neck cancer, evident by the complex maps and highly interconnected statements related to the experience of treatment. Implications for improving the quality of care delivered and care experience of head and cancer are discussed.

## Introduction

Head and neck tumours affect sensitive anatomical structures responsible for vital functions, such as speech, chewing and swallowing. Although treatments have shown promising oncological outcomes, they often have an impact on a person’s physical, psychological and social aspects of life [1,2]. Thus, healthcare providers (HCP) have been navigating the balance between cure and preserving quality of life [3], which in turn makes treatment planning for head and neck cancer (HNC) a complicated process. The process gets more challenging as, currently, patients are expected to be part of treatment planning and decision making. In this regard, it is valuable to understand patients’ perspectives around their experience with treatment, and how they might compare to the perspectives of HCPs.

Head and neck cancers are treated either by surgery, systematic therapy such as chemotherapy, radiation therapy, or some combination thereof [4]. In most cases, treatment can have distinct short and long-term toxicities affecting different aspects of an individual’s life. The use of multiple treatment modalities can further aggravate these toxicities [4–8]. Known structural and functional outcomes include disfigurement, scar formation, skin irritation, tissue fibrosis, lymphedema, hair loss, pain, and hyperalgesia. Oral intake and maintaining nutrition also can be affected by xerostomia, oral mucositis, trismus, dysphagia, dysgeusia, nausea, malnutrition, and weight loss. Communication might be impacted by either voice loss, speech disorder, hearing loss, tinnitus, vertigo, osteoradionecrosis, and fatigue [1,6,9,10]. With all these changes in physical functions, a deterioration in psychological wellbeing of individuals is anticipated [11]. People with history of HNC present with the highest levels of distress when compared to other cancers [12]. It is also known that anxiety, depression, fear of relapse, low self-esteem and embarrassment are expected [11,13]. Consequently, a decrease in social interactions is seen [1,11,13], highlighting the global impact of this type of cancer and its treatment on individual’s life.

Considering the impacts of treatment on a patient’s life, HCPs have been increasingly acknowledging quality of life assurance during treatment planning, along with ensuring cure and survival [3,14]. With the increasing focus on person-centred care, understanding patients’ perspectives became more important and including their perspective became necessary. In this regard, previous studies explored patients’ priorities and found that survival from cancer was their top priority [3,15,16], and that patients were inclined to accept treatment toxicities for survival [17]. Physical functions however, were still of high priority to patients. Swallowing for example, was ranked as the third highest priority following cure and survival in one study [18]. Thus, there appear to be competing priorities that still need to be more fully understood in the context of the experience of HNC. Recognizing the outcomes and priorities of those affected can be valuable to improving the quality of care and can inform future treatment planning.

To our knowledge, existing patient priorities are focused on functional outcomes, and little is known around perspectives on psychosocial and/or other aspects of outcomes [19]. The specific focus of priority studies on functional outcomes could be attributed to the fact that most priority measures were developed by investigators and presumed to include patients’ perspectives around the treatment of HNC. Yet, experiencing HNC diagnosis and treatment might include other valuable considerations that investigators might be missing when developing priority measures without initial input from patients. To help identify these considerations, it is imperative for patients to identify their priorities from their own experience.

### Purpose

The primary purpose of this study was to explore patients’ perspectives on HNC treatment and to actively identify their considerations by eliciting an exhaustive list of outcomes and considerations associated with their treatment experiences. We also sought to understand how these considerations are valued in terms of priority, and how they compare to HCPs’ priorities. Developing a comprehensive understanding of patients’ experience in this way will contribute to future treatment planning and enhance care experience for future patients.

## Methods

This exploratory, mixed-method study was ethically approved by the Health Research Ethics Board of Alberta: Cancer Committee (HREBA-CC-19-0012). All data collection was completed using CS Global Max™, a secure web-based platform for group concept mapping. Consent was indicated by overt action when participants submitted their responses online. Participants were able to withdraw responses up to the point of clicking the button “save and submit”. All responses were anonymous and therefore irretrievable once submitted.

### Participants

Convenience and snowball sampling methods were used to identify potentially eligible participants from our targeted population. Patients eligible for participation were adults 18 years or older, who were diagnosed with squamous cell HNC, were either receiving treatment or were post-treatment at the time of study enrollment, with treatments being delivered in Alberta, Canada. Patients with thyroid or salivary gland cancer (adenocarcinoma), and/or who did not start treatment, and/or patients with conditions that could compromise their ability to perform the required task in the study (e.g., dementia), were excluded from the study. Healthcare providers were individuals who regularly work with HNC patients in Alberta.

### Recruitment

Nine-hundred-and-eighty-five patients met the eligibility criteria within the Alberta Cancer Registry (ACR) database. The ACR directly mailed out study information packages that included the study information letter, ACR cover letter, and ACR information pamphlet to all potential participants. The study information letter included a direct link and a Quick Response (QR) code to scan and access the online platform and participate in the study. Patients and HCPs also were recruited through snowball recruitment methods including word of mouth. Posters (including the QR code) were displayed in public spaces such as hospitals and clinics where the target population receives consultation and treatment in Edmonton and Calgary. Recruitment took place between September and November 2019.

### Data collection

**Fig 1:**
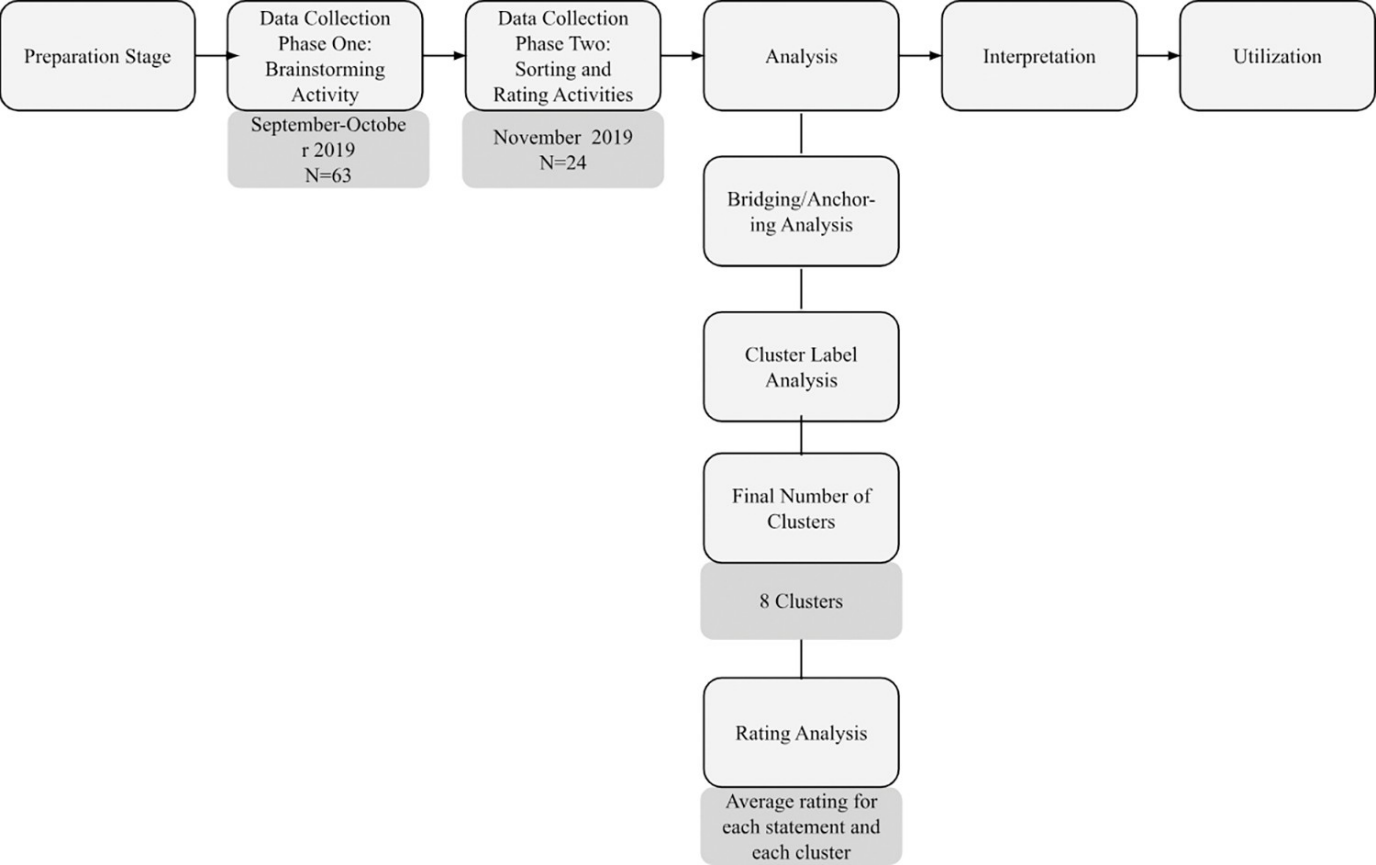
Group concept mapping process. This figure reflects the process of this study following the GCM process.

We used an exploratory, descriptive design using a mixed-method approach called Group Concept Mapping (GCM). The use of GCM was facilitated using the Concept Systems Global Max™ software (www.conceptsystems.com) as a web-based platform to collect and analyze data. A preparation stage prior to active data collection was undertaken to develop and test the prompt utilized in the data collection phase. Data collection in GCM is comprised of three activities: brainstorming, sorting, and rating [20]. In this study, data were collected in two phases; phase one (brainstorming activity) and phase two (sorting and rating activities) (Fig 1). These phases were conducted at separate time periods to reduce participant fatigue and attrition. In each phase, participants completed a questionnaire capturing demographic and clinical characteristics.

### Preparation for phase one

The research team identified the purpose of the study, target population, and developed and pilot tested the prompt used in the study. Developing the prompt is an essential step in GCM as it affects the outcome of the study [21]. The more accurate the prompt, the more accurate the elicited responses will be, and this can be determined by pilot testing the prompt on a group of representative target participants to ensure that it results in relevant statements [20]. The initial prompt was pilot tested on patients and HCPs with experience of HNC. The final crafted prompt was:

*“Important considerations throughout the entire experience of head and neck cancer are*..*”*

### Phase one

Phase one was comprised of an activity called brainstorming. Demographic questions were asked to capture the demographic and clinical characteristics of participants. In this activity, participants were instructed to provide as many statements as they could in response to the focused prompt (stated above). Brainstorming was estimated to take about 20–40 minutes to complete. This phase was open between September 16, 2019 and October 20, 2019.

Once brainstorming was closed, the statements collected underwent a structured and iterative synthesis process by research team, as described by Kane and Trochim [20]. All generated statements were reviewed following a structured synthesis process to remove redundancies from the brainstorming activity, in order to create a manageable number of concise and easily comprehended statements that would be carried forward to the sorting and rating activities. Previous literature supports the use of 100 statements or less to sufficiently cover the variety of thoughts while being manageable for participants in the sorting and rating activities.[20,22] In brainstorming, the quality of data depends on the data saturation rather than number of participants [20]. As stressed in the literature [20], we kept the edits at minimum, maintained the original meaning of the statement, and ensured that no ideas or thoughts have been removed during this process.

### Phase two

Demographic questions were asked to capture the demographic and clinical characteristics of participants. This phase included a sorting activity where each participant grouped statements together in terms of what they perceived to be similarity in theme. They then provided a label for each group that described a shared theme. This activity enabled us to better understand how participants perceive the interrelationship between statements. Participants received a “thank you” message upon submitting their final response in the sorting activity and a reminder to proceed and respond to the rating activity, with a reminder of the last day to submit the response for phase two.

Phase two also included the priority rating activity where participants rated each statement separately using a Likert-scale (1 to 5), with 1 being not important at all, and 5 is extremely important. This phase was estimated to take between 60 and 90 minutes in total. Participants could pause at any time and return to complete their response to the activity. The phase was open for three weeks, from November 9–30, 2019. Participants were provided with a link and login information. They were able to log in anytime within those three weeks.

### Data analysis

Data were analyzed using the (CS) Global Max™ software. The independent variable was participant group. Dependent variables included: statements, statement sorting results, and the rating of each statement. Covariates included: age, sex, marital status, education level, tumor site, stage of cancer at diagnosis, time since treatment, human papillomavirus (HPV) status, and treatment modality.

All statements from the brainstorming activity were converted to quantitative data by core mapping analysis. In this analysis, each statement was numbered and entered into a cross-correlational matrix. From there, a tally of the number of times two statements occurred together was created. A series of analyses took place within the software after the creation of this matrix [20]. The reader is referred to (S6 Appendix) for a detailed description of these analyses.

Nonmetric multidimensional scaling of the matrix was performed to site each statement as a (x,y) point on the point map and depict the relative distance between statements. Stress value was used to assess the goodness-of-fit of the resulting map, with lower values indicating better fit, with an average of 0.285 and range (0.205–0.365) being suggested in the literature as target values [20]. The (x,y) points on the point map underwent a hierarchical cluster analysis that identified the representation of how statements were grouped together in terms of similarity. Each possible cluster map with clusters 6–10 was examined using the cluster and statement bridging values, along with interpretation of cluster contents to identify the final number of clusters that best fit the results. The multidimensional maps generated by the software were the visual representation of the project’s data and finding. They depicted how the participants perceived the statements in terms of similarity, and their importance,

## Results

### Phase one

A total of 63 participants (n = 59 patients, n = 4 HCPs) completed phase one. Detailed participant demographic and clinical characteristics are reported in Table 1. Two hundred fifty statements were generated. Data variety was ensured by the number of statements generated and a wide range of perspectives captured. For a successful synthesized set of statements, the number of statements was reduced to 94 statements after the synthesis process without comprising any ideas generated. This number falls under the average reported number of statements found in the literature of 96 statements (Rosas & Kane, 2012). Most of the statements were semantically and syntactically preserved from the original response gathered.

**Table 1:** Demographic and clinical characteristics of participants.

Characteristics	Responses in phase one (n = 63)		Responses in phase two (n = 24)	
Role n (%)	Patients 59 (93.65)	Healthcare Providers 4 (6.35)	Patients 15 (62.50)	Healthcare provider (9 (37.50)
Age range	44–71	30–54	31–69	24–71
Sex n (%)				
Male	41 (69.49)	1 (25)	11(73.33)	2 (22.22)
Female	18 (30.50)	3 (75)	4 (26.66)	7 (77.77)
Marital status n (%)				
Single	3 (5.08)	0 (0.00)	2 (13.33)	1 (11.11)
Married or in a domestic partnership	50 (84.74)	4 (100)	11 (73.33)	8 (88.88)
Widowed	0 (0.00)	0 (0.00)	0 (0.00)	0 (0.00)
Divorced or separated	6 (10.16)	0 (0.00)	2 (1.33)	0 (0.00)
Education level n (%)				
Less than high school degree	1 (1.69)	0 (0.00)	1 (6.66)	0 (0.00)
High school degree or equivalent	20 (33.89)	1 (25	4 (26.66)	0 (0.00)
Associate degree	15 (25.42)	0 (0.00)	3 (20)	2 (22.22)
Bachelor degree	16 (27.11)	0 (0.00)	5 (33.33)	1 (11.11)
Graduate degree	11 (18.64)	3 (75)	2 (13.33)	6 (66.66)
Tumor site (multiple options apply) n (%)				
Nasal cavity and paranasal sinus*	0 (0.00)		0 (0.00)	
Oral cavity (mouth, lips, gum, tongue)*	39 (56.52)		11 (61.11)	
Pharynx (throat)*	6 (8.70)		3 (16.67)	
Larynx (voice box)*	2 (2.90)		1 (5.56)	
Other*	22 (31.88)		3 (16.67)	
Stage at diagnosis n (%)				
Early stage	25 (43.10)		6 (40.00)	
Advanced stage	29 (50.00)		7 (46.67)	
Not known	4 (6.90)		2 (13.33)	
Time since treatment n (%)				
Currently undergoing treatment	2 (3.45)		0 (0.00)	
Less than 6 months post-treatment	0 (0.00)		0 (0.00)	
6–12 months post-treatment	1 (1.72)		0 (0.00)	
1–2 years post-treatment	8 (13.79)		1 (6.67)	
2–5 years post treatment	15 (25.86)		4 (26.67)	
More than 5 years post treatment	32 (55.17)		10 (66.67)	
Treatment type (multiple options apply) n (%)				
Surgery*	38 (29.92)		12 (34.29)	
Chemotherapy*	32 (29.92)		8 (22.86)	
Radiation therapy*	49 (38.58)		12 (34.29)	
Immunotherapy*	2 (1.57)		1 (2.86)	
Targeted therapy *	1 (0.79)		0 (0)	
Other *	5 (3.94)		2 (5.71)	
Healthcare provider specialty n (%)				
Physician		0 (0.00)		
Allied health clinician		4 (100)		
Clinical support		0 (0.00)		
HPV n (%)**			6 (28.57)	
HPV+			5 (23.81)	
HPV-			2 (9.52)	
Not known			8 (38.10)	
Not applicable				

*Denote multiple options apply. Participants were able to choose more than one tumor site and/or treatment type as it applies to their case.

**Denote identified subjectively by participants.

### Phase two

A total of twenty-four people (could be different than those participated in phase one) participated in phase two (n = 15 patients, n = 9 HCPs) and completed either sorting and/or rating activities. Details of participants’ demographics in Table 1. Based on the literature, 20–30 sorters in total reflect the diversity of perspectives and is sufficient to reduce the stress value and provide reliable maps [23].

#### Sorting

In phase two, 22 out of the 24 participants completed the response sorting task. One participant’s sorting responses were removed because the sorting was based on what the outcome meant to the individual (e.g., not an issue for me, not a serious issue for me, major concerns) rather than categorizing statements into groups based on their conceptual similarities. Thus, 21 sorters’ responses were identified and eligible for core analysis.

**Fig 2:**
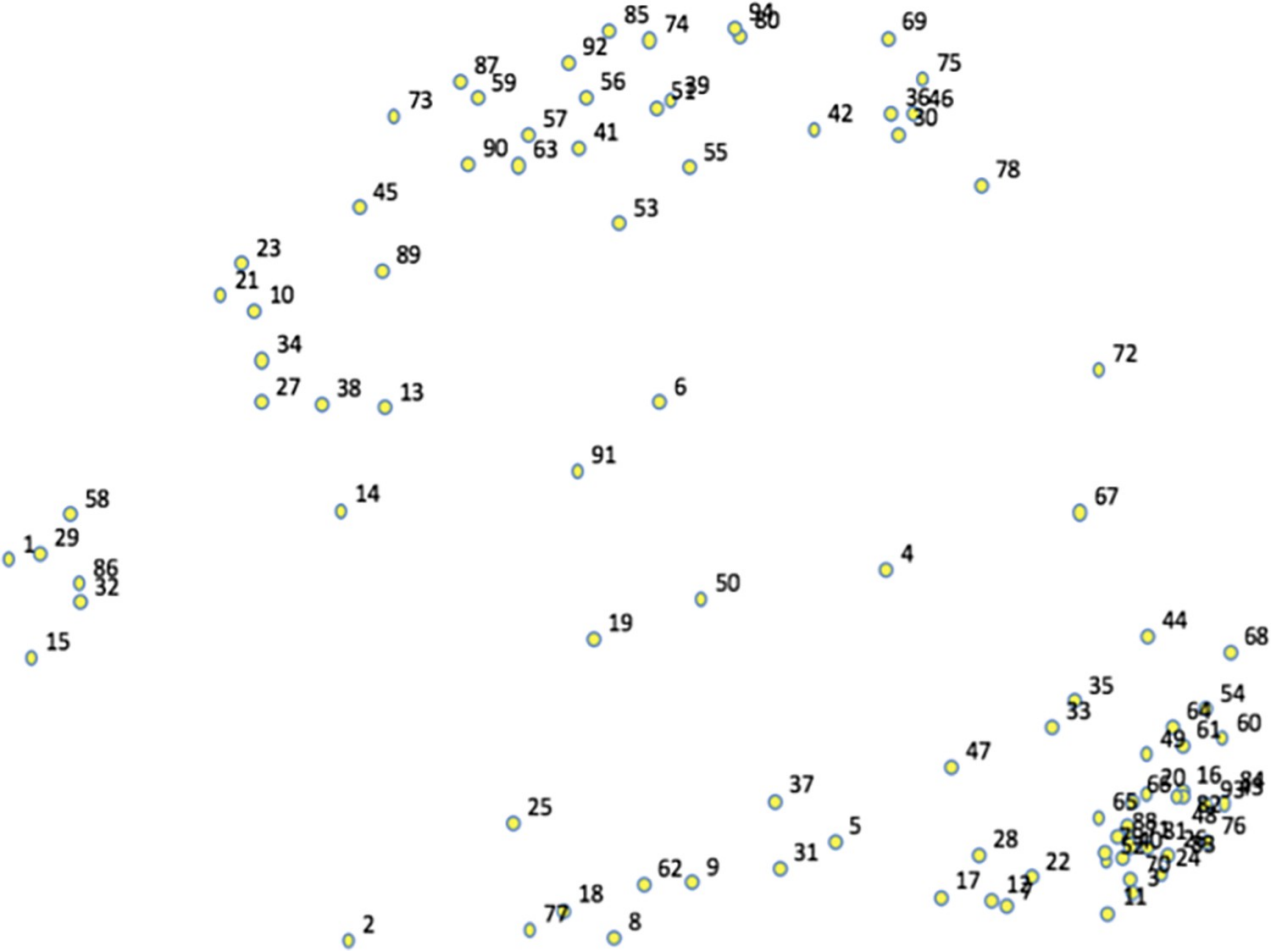
Point map. This figure illustrates the 94 statements in (x,y) points. Areas with several points close together indicate the higher agreement upon participants that these statements shared similar theme.

**Fig 3:**
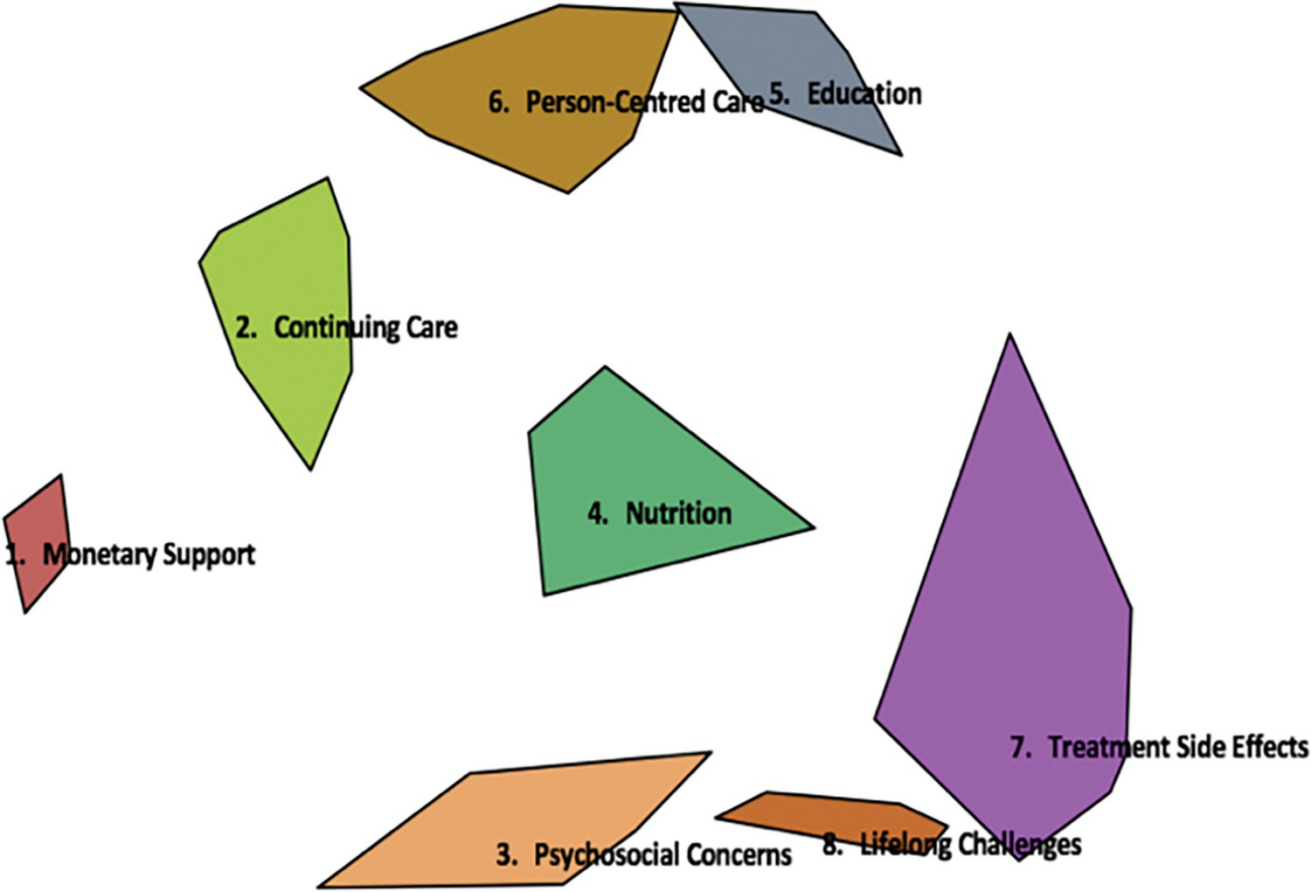
Cluster map. This figure depicts the eight clusters of statements. Smaller clusters are more focused in concept while larger clusters are relatively heterogeneous.

The multidimensional scaling (MDS) analysis of sorting data generated the point map from which all further analyses were based (Fig 2). All 94 statements are represented on this map, as each point represents a statement/outcome. A final MDS stress value of 0.2213 was obtained. Following that, a cluster analysis of the 94 (x,y) point map was examined via cluster bridging values along with the cluster replay map and the research team’s judgement to identify the final number of clusters and for or our purpose, we considered an eight-cluster structure as a best fit to our results (Fig 3). Table 2 provides description of each cluster.

**Table 2:** Cluster descriptions.

Cluster label	Number of statements	General focus of cluster
1 ‘Monetary Support’	6	This small cluster focused on accessibility of treatment and health-related sources especially for out of town patients. It also covered the financial aspect/burden of treatment.
2 ‘Continuing Care’	10	Statements within this cluster reflected continuing care after initial treatment including long-term follow ups, routine checkup appointments, and allied health services.
3 ‘Psychosocial Concerns’	8	This cluster represented the psychological outcomes of treatment and the influence the treatment has on social life and interaction including within the work environment.
4 ‘Nutrition’	5	Statements included factors related to nutrition: the quality of feeding tube food, proper fit of dentures and maintaining a healthy diet. Survival was also included in this cluster.
5 ‘Education’	8	This cluster focused on all types of information patients need throughout the experience of head and neck cancer, information around diagnosis, disease, treatment plan and treatment options, expected outcomes, and possible impacts of treatment.
6 ‘Person-Centred Care’	16	The content of this cluster covered all aspects of support to patients and family before, during, and after treatment, it also contained statements around the quality of healthcare provided in terms of the healthcare team’s engagement, communication and tracking patients’ progress.
7 ‘Treatment Side Effects’	34	These statements reflected mainly the physical and functional impacts of treatment.
8 ‘Lifelong Challenges’	7	Statements in this cluster reflected physical and functional impacts of treatment that are of a persistent nature or with uncertain interventions available for them.

The central location of cluster 4 ‘Nutrition’ reflects the relationship of the statements in this cluster with different aspects and statements from other clusters. Survival might be located at this area of the map, not because of how much it is connected to nearby statements of ‘Nutrition’ but rather because it might be related to several other areas and clusters on the map.

#### Rating

Twenty-three out of the 24 participants completed rating for all 94 statements. The average rating was computed for each statement and for each cluster of statements. For both groups combined, the top five ranked singular statements in terms of importance (i.e., average ranked scores out of 5) identified at a statement level were ‘*promptness of treatment*’ (4.91), ‘*promptness of diagnosis*’ (4.86), ‘*knowledgeable and experienced healthcare providers in head and neck cancer*’ (4.82), ‘*survival*’ (4.68), and ‘*clear detailed upfront information of the case and treatment plan*’ (4.64).

**Fig 4:**
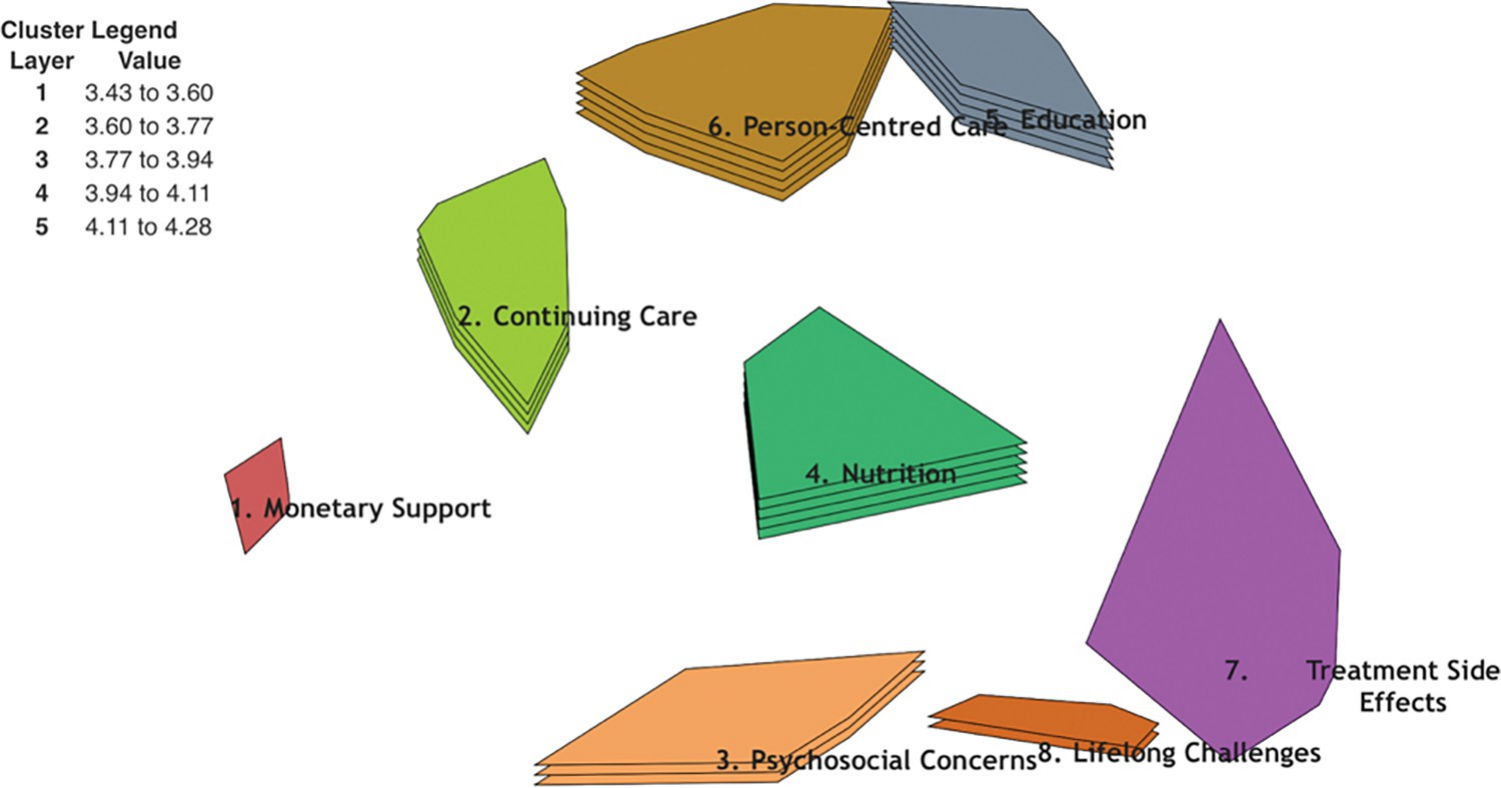
Cluster rating map. This figure illustrates the clusters in layers reflecting average rating. The more layers, the higher the average rating is given to the cluster in terms of priority.

The highest rated clusters for both groups combined in terms of importance were ‘Education’, with an average rating of (4.28), ‘Person-Centred Care’ with an average rating of (4.20), ‘Nutrition’ with an average rating of (4.15), and ‘Continuing Care’ with an average rating of (4.08). The average rating values of the clusters are plotted in the cluster rating map (Fig 4).

**Fig 5:**
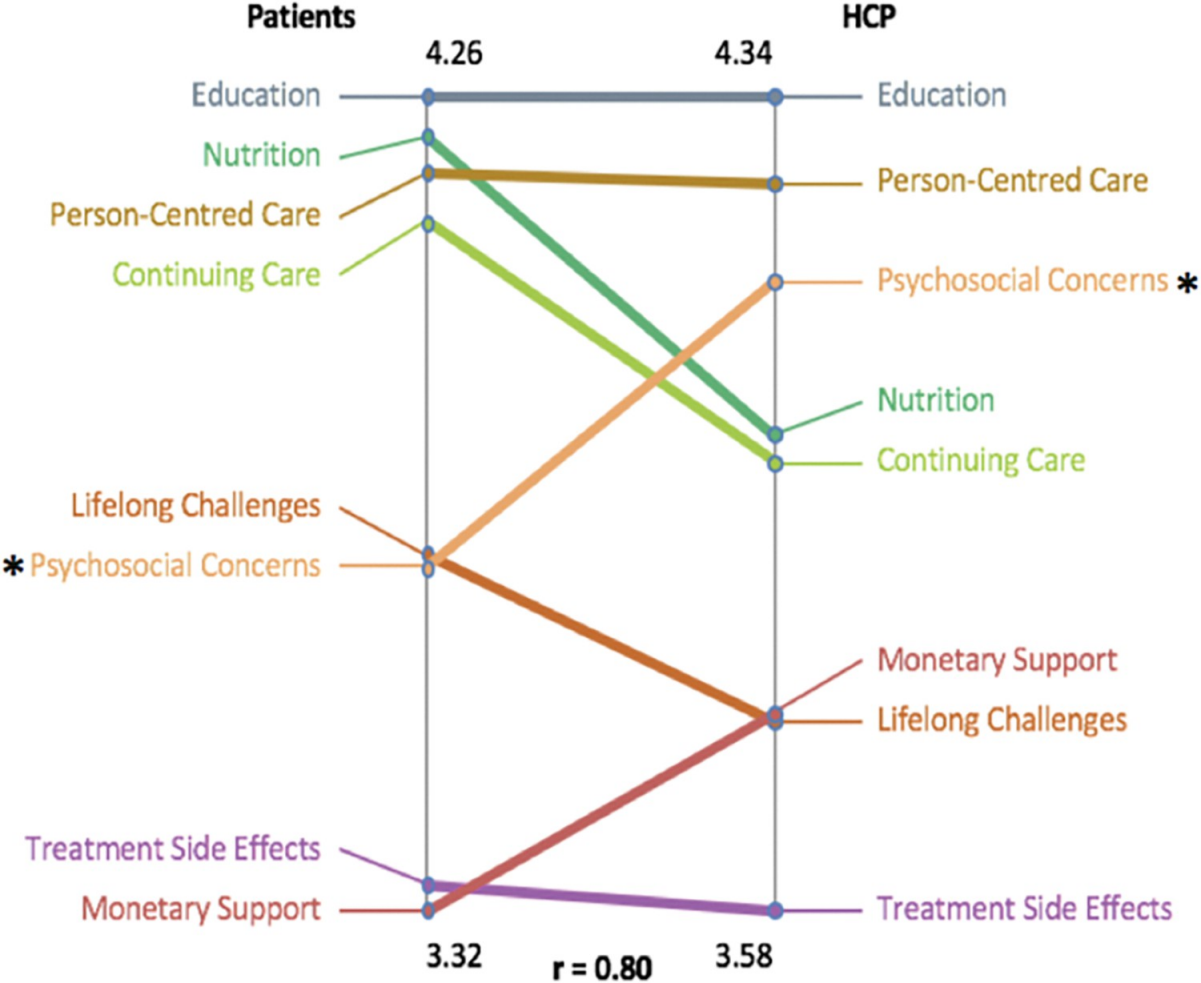
Pattern match: Patients and Healthcare Providers (HCP). This figure illustrates the average rating agreement between patients and HCP in terms of importance. *denote statistically significant difference.

The pattern match (Fig 5) represents the level of agreement on the importance of outcomes (clusters) between patients and HCPs. Overall, there was a strong correlation between patient and HCPs’ ratings (r = 0.80). However, although ‘Psychosocial Concerns’ was the third highest-ranked priority for HCPs, it was the fifth most important consideration for patients. This difference was found to be statistically significant (t (14) = 2.76, p <0.02).

## Discussion

In this study, we aimed to explore the HNC treatment experience and priorities from the perspective of those affected. Our first objective was that participants identify treatment outcomes and considerations in a brainstorming activity that would then be rated in terms of priority. As a result, our findings from the brainstorming activity revealed treatment experience-related considerations that were reported previously in the literature, and others that were not investigated previously in terms of their priority. We also measured priorities for patients and HCPs at a statement level, revealing the following top priorities: timely diagnosis and treatment, having HCPs experienced with HNC, survival, and having clear upfront information on treatment. We then measured priorities at a cluster level to understand how general themes might differ in priority. The ‘Education’ cluster was the highest rated cluster. Furthermore, we explored the relationship between patients’ and HCPs’priorities to help us understand how to enhance future treatment planning and care experience. Findings revealed an alignment of most priorities between the two groups, except for a significant difference of priority rating in the ‘Psychosocial Concerns’ context. The findings of this study bring new information to the literature that may suggest that previous consideration of priorities has been narrow in focus. Specifically, in the present study, when priorities were identified from patients’ perspectives, they were holistic of the care experience and not specific to treatment-related outcomes or side effects. Thus, while treatment side-effects are a reality of the condition of HNC, they should be considered in a wider context of the health care experience.

### Patient-reported outcomes in the health care context

In examining treatment outcomes and priorities, previous studies used measures developed by investigators [24,25]. It is now well-recognized in clinical practice and research that patients’ perspectives are essential from the outset to fully understand their care experience and assess treatment options [24,25]. In our study, we wanted to explore if there are valuable priorities that are missed by investigators and which can only be identified by individuals who experienced HNC treatment. To that end, patients and HCPs in our study actively identified a holistic set of statements/ considerations related to their treatment experience, and were refined from a brainstorming activity. These included statements not only related to treatment outcomes but also to healthcare-related, reflecting the global and complicated impact of this illness. Treatment outcomes reported in the literature that also were identified in the present study included: survival [3,15,26]; pain and functional outcomes [3,15,26,27]; physical alterations and psychological outcomes [1,2]; social impact of treatment [1,11,13]; financial challenges [28,29]; support and involvement in decision-making [30]. Taking higher priority, however, were healthcare-related considerations centered around the care experience including accessibility to health services for those in rural communities; care services after primary intervention; psychosocial support before, during, and after treatment; HCPs’ knowledge; and patient and family education. The resulting maps provided visuals on how outcomes related to healthcare experience and services were more important for both groups than treatment-related outcomes and side-effects. This might reflect how meaningful it is for people with history of HNC to have a positive care experience, and might reflect the importance that education and support have on their care experience.

### Priority ranking of healthcare-related considerations

As we explored priority ranking among both groups, we found that promptness of treatment, education, and support were the most valued considerations.

### Timeliness of care

At a *statement* level, promptness of treatment was ranked the highest for both patients and HCPs. Despite recognizing the need for prompt HNC intervention in ensuring optimal outcomes, delays in delivering care for HNC are frequently reported [31,32]. These delays ultimately affect functional outcomes [32,33] and mortality [32,34]. Barriers to timely treatments still exist [35], and successful strategies to facilitate the promptness of HNC treatment remains undetermined [32]. Within the context of planning and providing timely treatment, delivering information around treatment plan and outcomes also was highly valuable for our participants.

### Education

While recognizing promptness of treatment is a priority, participants also identified an important consideration to their treatment experience, which was education. Education, as a theme or cluster, was the highest ranked among our participants. The results of our study further showed that there were two categories of information that patients and HCPs identified as critical to the experience of HNC treatment. The first was providing education on the treatment options and what the treatment will entail, and the second was on the potential long-term side effects that patients may encounter and how to manage and cope with these effects. The value of these types of information has been substantiated in the literature in several studies as it highlighted the impact on overall outcomes [36]. Besides acknowledging the value of such information, considerations around a patient’s emotional readiness to comprehend information is also critical.

Patients have different preferences regarding when to receive information around their treatment. In previous research, patients have stressed the importance of receiving thorough information on all expected outcomes before initiating treatment to better anticipate and prepare for possible side effects [36,37]. While some patients preferred to know everything at the time of diagnosis, others felt it was difficult to comprehend information on side effects when provided right after diagnosis [37], and others reported difficulty remembering information provided before treatment sessions regarding their treatment plan [37]. Consequently, patients reported the value of having an accessible resource for information. In the age of the internet, patients seek health information related to symptoms, etiologies and treatment options online and even try to educate themselves in interpreting test results [38]. Such health behaviors pose a risk that patients may rely on nonmedical sources to inform their decision-making. This highlights the need for trustworthy and informed sources to provide appropriate information to patients and their families, in a timely manner [30,36,39] and aligns with the priority found in our study *‘providing an accessible resource/tool for information and common questions*’. Our findings point to the need for establishing strategies to support communication, deliver information continuously, and to outline pathways for following-up with patients throughout their treatment experience.

### Person-centred care

Developing strategies for communication should consider that successful patient-clinician interaction is a part of the support that patients need to be involved in their care. This in turn is the cornerstone of a person-centered approach of care. The ‘Person-Centred Care’ cluster in the present study included statements around involving the patient and their families in care decisions, support, and knowledge.

Participants in our study reported that *‘being involved in decision-making*’ and ‘*keeping the family informed’* are important when planning treatment for HNC. Patients generally consider it highly important to include their families and friends in the decision-making process and consider their opinions and concerns throughout the treatment process [30]. Yet, not having sufficient information on the treatment options makes it difficult to be actively involved in decision-making [40]. Thus, to be able to discuss and share thoughts around decisions related to care, patients need to be educated on the nature of their illness, their care options, prognosis and expected outcomes, and to be supported to share thoughts and concerns during their care process.

As a form of support, patients appreciate HCPs showing empathy and care, as well as those who communicate actively with them [36]. *‘Communication with healthcare providers*’ and *‘Engaged healthcare providers with compassionate care*’ were high priorities in the ‘person-centred care’ cluster in this study. Patients need to communicate effectively with HCPs to be able to make decisions. They also need to be comfortable to provide feedback on treatment options, outcomes and share their concerns following treatment, which may include physical, functional and psychosocial impacts.

### Psychosocial concerns

The psychosocial impacts following cancer treatment were identified by our participants as an area to be considered when planning treatment. Although it was the third priority for HCPs, it was only the fifth important consideration for patients. Different interpretations might justify this significant difference of rating. Healthcare providers have access to research focused on psychosocial impact of treatment. One study, for instance, reported that two out of the four highest suicidal populations with cancer are those with history of oral cavity and laryngeal cancers [41]. The continuous exposure to similar reports might have an influence on HCPs’ perspective. Moreover, the topic of clinical empathy has been getting attention in recent years, and training to improve skills of empathy in HCPs has been supported [42]. This focus is derived from the positive relationship found between clinical empathy and improved care. It also was reported that with empathy, patient-clinician interaction is enhanced and patients therefore become more open to share their worries and concerns [42]. The focus on empathy and the patient-clincian interaction could explain why HCPs perceive psychosocial concerns as a high priority [40].

Psychosocial impacts of cancer treatment are highly prevalent in individuals with HNC and these outcomes were important for patients in our study. Yet, it was interesting to see that continuing care after treatment was of a higher priority to them, reducing the ranking of psychosocial concerns. This might reflect how patients focused their priority rating on accessibility to care, care and support services, education and resources, more than anatomical, functional, and psychosocial treatment impacts. This significant difference in priority requires further investigation in future work.

### Limitations

There are several limitations to the study that may limit the generalizability of the findings. Although the sample was sufficient to produce valid and reliable data in GCM, it was small to be generalizable for all patients and HCPs who have experience with HNC. The small sample of participants with different types of HNC limits the ability to perform sub-analysis based on each type. Participants in this study experienced treatment only from the province of Alberta and because healthcare is provincial in Canada, our findings from Albertans may not be representative of patients in other geographical regions. A larger study can consider the priorities of HNC patients globally. This would allow for further investigation of underlying factors causing the differences in priorities. Other limitation related to study design is noted in the wording of the rating question, the question stated to rate statements in terms of “importance”, which can be perceived differently than “priority” and thus perhaps have caused participants to rate statements differently.

Although one of the objectives of the study was to compare the priorities of patients and HCPs, there might be a selection-bias where a higher number of patients participated in the rating activity compared to HCPs. This although did not affect the significance of the analysis and findings, it may present some bias toward the conclusion. Non-respondent bias could be present in this study, given that patients with psychosocial may not be as motivated to participate in a study. Thus, cautions should be taken with generalization of findings and conclusions might take this into consideration.

Despite the fact that this GCM facilitated the active identification of treatment considerations from participants and allowed the capture of multiple perspectives, it does not provide us with a deep understanding of why some outcomes are of a higher priority than others. Future studies might consider conducting qualitative interviews or focus groups to further understand the participants’ priorities. Of particular importance would be to understand why education and person-centred care are higher priorities than side-effects. Are these priorities unmet? Or otherwise met and believed to be factors for a successful experience? Further research may investigate details of priorities in each phase: diagnosis, treatment, and transitioning to rehabilitation.

Another study limitation was the low response in the online method used. Although online methods of GCM have certain benefits including access to many participants, a low response rate has been addressed in the literature and noted in our study [20]. Lastly, one of the outcomes extracted from brainstorming activity (i.e., “tongue pain”) was accidently duplicated in the synthesized set, and participants had to sort and rate this statement twice. When the software experts were consulted, the duplication issue was found to be somehow frequent, and since the duplicated statement was found together in the same cluster after analysis, no negative effect is estimated.

## Conclusion

Person-centred care requires an understanding of patient needs and priorities. Based on the results of our study on priorities, multiple considerations should be taken into account to improve the HNC care experience. The perspective of patients and HCPs’ priorities were largely in agreement. Timely care, education, and person-centred care were identified as the most meaningful considerations for future treatment planning.

## Supporting information

S1 Appendix Brainstorming activity instructions. (DOCX)

S2 Appendix Sorting activity instructions. (DOCX)

S3 Appendix Rating activity instructions. (DOCX)

S4 Appendix List of final statements included in phase two. (DOCX)

S5 Appendix List of clusters and statements (Ascending Rating Value). (DOCX)

S6 Appendix Analysis process of group concept mapping. (DOCX)

S7 Appendix Consent. (DOCX)

S8 Appendix Participant questions. (CSV)

S9 Appendix Full set of statements. (PDF)

S10 Appendix Sorting data. (CSV)

S11 Appendix Rating data. (CSV)

## References

[1] List MA, Bilir SP. Functional outcomes in head and neck cancer. 2004;14(2):178–189. doi: 10.1053/j.semradonc.2003.12.008 15095263

[2] You EL, Henry M, Zeitouni AG. Human papillomavirus-associated oropharyngeal cancer: Review of current evidence and management. *Current oncology (Toronto*, *Ont*.*)*. 2019;26(2):119–123. doi: 10.3747/co.26.4819 31043814 PMC6476447

[3] Gill SS, Frew J, Fry A, et al. Priorities for the head and neck cancer patient, their companion and members of the multidisciplinary team and decision regret. *Clinical oncology*. 2011. doi: 10.1016/j.clon.2011.03.014 21550217

[4] Machtay M, Moughan J, Trotti A, et al. Factors associated with severe late toxicity after concurrent chemoradiation for locally advanced head and neck cancer: An RTOG analysis. *JCO*. 2008;26(21):3582–3589. doi: 10.1200/JCO.2007.14.8841 18559875 PMC4911537

[5] Strojan P, Hutcheson KA, Eisbruch A, et al. Treatment of late sequelae after radiotherapy for head and neck cancer. *Cancer Treat Rev*. 2017;59:79–92. doi: 10.1016/j.ctrv.2017.07.003 28759822 PMC5902026

[6] Dzioba A, Aalto D, Papadopoulos-Nydam G, et al. Functional and quality of life outcomes after partial glossectomy: A multi-institutional longitudinal study of the head and neck research network. *Journal of Otolaryngology—Head &* *Neck Surgery*. 2017;46(1):1–11. doi: 10.1186/s40463-017-0234-y 28870248 PMC5583999

[7] Epstein JB, Wilkie DJ, Fischer DJ, Kim Y, Villines D. Neuropathic and nociceptive pain in head and neck cancer patients receiving radiation therapy. *Head &* *Neck Oncology*. 2009;1(1):26. doi: 10.1186/1758-3284-1-26 19594943 PMC2717963

[8] Landier W. Ototoxicity and cancer therapy. *Cancer*. 2016;122(11):1647–1658. doi: 10.1002/cncr.29779 26859792

[9] Mayo Foundation for Medical Education and Research. Cancer fatigue: Why it occurs and how to cope. Mayo Clinic Web site. https://www.mayoclinic.org/diseases-conditions/cancer/in-depth/cancer-fatigue/art-20047709. Updated 2018. Accessed March 8, 2020.

[10] Gegechkori N, Haines L, Lin JJ. Long-term and latent side effects of specific cancer types. *Med Clin North Am*. 2017;101(6):1053–1073. https://pubmed.ncbi.nlm.nih.gov/28992854 https://www.ncbi.nlm.nih.gov/pmc/articles/PMC5777532/. doi: 10.1016/j.mcna.2017.06.003 28992854 PMC5777532

[11] Nguyen NP, Frank C, Moltz CC, et al. Impact of dysphagia on quality of life after treatment of head-and-neck cancer. *International Journal of Radiation Oncology*, *Biology*, *Physics*. 2005;61(3):772–778. http://www.sciencedirect.com/science/article/pii/S0360301604010740. doi: 10.1016/j.ijrobp.2004.06.017 15708256

[12] Mehnert A, Brähler E, Faller H, et al. Four-week prevalence of mental disorders in patients with cancer across major tumor entities. *Journal of Clinical Oncology*. 2014;32(31):3540–3546. doi: 10.1200/JCO.2014.56.0086 25287821

[13] Ward EC, Bishop B, Frisby J, Stevens M. Swallowing outcomes following laryngectomy and pharyngolaryngectomy. *Archives of Otolaryngology–Head &* *Neck Surgery*. 2002;128(2):181–186. doi: 10.1001/archotol.128.2.181 11843728

[14] Demez PH, Moreau PR. Perception of head and neck cancer quality of life within the medical world: A multicultural study. *Head Neck*. 2009;31(8):1056–1067. doi: 10.1002/hed.21069 19340871

[15] Tschiesner U, Sabariego C, Linseisen E, et al. Priorities of head and neck cancer patients: A patient survey based on the brief ICF core set for HNC. *Eur Arch Otorhinolaryngol*. 2013;270(12):3133–3142. http://www.ncbi.nlm.nih.gov/pubmed/23543319. doi: 10.1007/s00405-013-2446-8 23543319

[16] Badr H, Rosenthal DI, Milbury K, et al. Do the treatment outcome priorities of head and neck cancer patients change after undergoing radiation treatment? *JCO*. 2010;28(15):5599.

[17] Windon MJ, D’Souza G, Fakhry C. Treatment preferences in human papillomavirus-associated oropharyngeal cancer. *Future oncology (London*, *England)*. 2018;14(24):2521–2530. https://www.ncbi.nlm.nih.gov/pubmed/30265132 https://www.ncbi.nlm.nih.gov/pmc/articles/PMC6275561/. doi: 10.2217/fon-2018-0063 30265132 PMC6275561

[18] Windon MJ D’Souza G, Faraji F, et al. Priorities, concerns, and regret among patients with head and neck cancer. *Cancer*. 2019;125(8):1281–1289. doi: 10.1002/cncr.31920 30645761 PMC6443481

[19] Cracchiolo JR, Klassen AF, Young-Afat D, et al. Leveraging patient-reported outcomes data to inform oncology clinical decision making: Introducing the FACE-Q head and neck cancer module. *Cancer*. 2019;125(6):863–872. doi: 10.1002/cncr.31900 30500993 PMC6403001

[20] Kane M, Trochim WMK. *Concept mapping for planning and evaluation*. SAGE Publications; 2007. https://books.google.ca/books?id=Vpb30j-RXqoC.

[21] Sjodahl Hammarlund C, Nilsson MH, Hagell P. Measuring outcomes in parkinson’s disease: A multi-perspective concept mapping study. *Qual Life Res*. 2012;21(3):453–463. doi: 10.1007/s11136-011-9995-3 21870190

[22] Trochim WMK. An introduction to concept mapping for planning and evaluation. *Evaluation and Program Planning*. 1989;12(1):1–16. http://www.sciencedirect.com/science/article/pii/0149718989900165. doi: 10.1016/0149-7189(89)90016-5

[23] Rosas SR, Kane M. Quality and rigor of the concept mapping methodology: A pooled study analysis. *Eval Program Plann*. 2012;35(2):236–245. doi: 10.1016/j.evalprogplan.2011.10.003 22221889

[24] Mendez A, Seikaly H, Eurich D, et al. Development of a patient-centered functional outcomes questionnaire in head and neck cancer. *JAMA Otolaryngol Head Neck Surg*. 2020;146(5):437–443.. Accessed 7/13/2021. doi: 10.1001/jamaoto.2019.4788 32271362 PMC7146527

[25] Ahmed S, Berzon RA, Revicki DA, et al. The use of patient-reported outcomes (PRO) within comparative effectiveness research: Implications for clinical practice and health care policy. *Med Care*. 2012:1060–1070. doi: 10.1097/MLR.0b013e318268aaff 22922434

[26] Badr H, Rosenthal DI, Milbury K, et al. Do the treatment outcome priorities of head and neck cancer patients change after undergoing radiation treatment? *JCO*. 2010;28(15):5599.

[27] Wilson JA, Carding PN, Patterson JM. Dysphagia after nonsurgical head and neck cancer treatment. *Otolaryngology–Head and Neck Surgery*. 2011;145(5):767–771. http://journals.sagepub.com/doi/full/10.1177/0194599811414506. 21746839 10.1177/0194599811414506

[28] Morales CZ, McDowell L, Lisy K, Piper A, Jefford M. Return to work in survivors of human Papillomavirus–Associated oropharyngeal cancer: An australian experience. *International Journal of Radiation Oncology*Biology*Physics*. 2020;106(1):146–156. http://www.sciencedirect.com/science/article/pii/S0360301619337514. doi: 10.1016/j.ijrobp.2019.09.001 31521718

[29] Taylor JC, Terrell JE, Ronis DL, et al. Disability in patients with head and neck cancer. *Arch Otolaryngol Head Neck Surg*. 2004;130(6):764–769. Accessed 3/31/2020. doi: 10.1001/archotol.130.6.764 15210560

[30] Bisschop JAS, Kloosterman FR, van Leijen-Zeelenberg JE, Huismans GW, Kremer B, Kross KW. Experiences and preferences of patients visiting a head and neck oncology outpatient clinic: A qualitative study. *European Archives of Oto-Rhino-Laryngology*. 2017. doi: 10.1007/s00405-017-4453-7 28132135 PMC5383674

[31] Graboyes EM, Garrett-Mayer E, Sharma AK, Lentsch EJ, Day TA. Adherence to national comprehensive cancer network guidelines for time to initiation of postoperative radiation therapy for patients with head and neck cancer. *Cancer*. 2017;123(14):2651–2660. doi: 10.1002/cncr.30651 28241092

[32] Graboyes EM, Kompelli AR, Neskey DM, et al. Association of treatment delays with survival for patients with head and neck cancer: A systematic review. *JAMA Otolaryngol Head Neck Surg*. 2019;145(2):166–177. Accessed 6/8/2020. doi: 10.1001/jamaoto.2018.2716 30383146 PMC6494704

[33] Xiao R, Ward MC, Yang K, et al. Increased pathologic upstaging with rising time to treatment initiation for head and neck cancer: A mechanism for increased mortality. *Cancer*. 2018;124(7):1400–1414. doi: 10.1002/cncr.31213 29315499

[34] Guttmann DM, Kobie J, Grover S, et al. National disparities in treatment package time for resected locally advanced head and neck cancer and impact on overall survival. *Head Neck*. 2018;40(6):1147–1155. doi: 10.1002/hed.25091 29394465

[35] Ho AS, Kim S, Tighiouart M, et al. Quantitative survival impact of composite treatment delays in head and neck cancer. *Cancer*. 2018;124(15):3154–3162. doi: 10.1002/cncr.31533 29742280 PMC6097917

[36] Checklin M, Bain J, Bath L, Lethbridge K. Patients’ perspectives on what makes a better care experience while undergoing treatment for oropharyngeal dysphagia secondary to head and neck cancer. *Dysphagia*. 2019. doi: 10.1007/s00455-019-10077-y 31748827

[37] Brockbank S, Miller N, Owen S, Patterson JM. Pretreatment information on dysphagia: Exploring the views of head and neck cancer patients. *J Pain Symptom Manage*. 2015;49(1):89–97. doi: 10.1016/j.jpainsymman.2014.04.014 24929028

[38] Dodd RH, Forster AS, Marlow LAV, Waller J. Psychosocial impact of human papillomavirus-related head and neck cancer on patients and their partners: A qualitative interview study. *Eur J Cancer Care*. 2019;28(2):e12999. doi: 10.1111/ecc.12999 30677190 PMC6559265

[39] Llewellyn C, McGurk M, Weinman J. Striking the right balance: A qualitative pilot study examining the role of information on the development of expectations in patients treated for head and neck cancer. *Psychology*, *Health & Medicine*. 2005;10(2):180–193. http://www.tandfonline.com/doi/abs/10.1080/1354850042000326593.

[40] Edwards D. Head and neck cancer services: Views of patients, their families and professionals. *British Journal of Oral & Maxillofacial Surgery*. 1998;36(2):99–102. http://www.sciencedirect.com/science/article/pii/S0266435698901759. doi: 10.1016/s0266-4356(98)90175-9 9643593

[41] Misono S, Weiss NS, Fann JR, Redman M, Yueh B. Incidence of suicide in persons with cancer. *Journal of clinical oncology*: *official journal of the American Society of Clinical Oncology*. 2008;26(29):4731–4738. https://pubmed.ncbi.nlm.nih.gov/18695257 https://www.ncbi.nlm.nih.gov/pmc/articles/PMC2653137/. doi: 10.1200/JCO.2007.13.8941 18695257 PMC2653137

[42] Hardy C. Empathizing with patients: The role of interaction and narratives in providing better patient care. *Medicine*, *Health Care and Philosophy*. 2017;20(2):237–248. doi: 10.1007/s11019-016-9746-x 27796726

