# Supplementary material for: Head and neck cancer treatment outcome priorities: A multi-perspective concept mapping study
Source: PLoS One. 2023 Nov 30;18(11):e0294712. doi: 10.1371/journal.pone.0294712 (PMC10688684; doi:10.1371/journal.pone.0294712)

**S1 Appendix**

**Brainstorming Activity Instructions**

In the text box below, type a statement that completes or answers the focus prompt.

- You may add **as many** statements as you wish.
- Please keep each statement **brief**, just **one** thought.
- Select “add this statement” **after each** statement or idea. Your statement will be then saved and added to the list of collected statements at the bottom of the page.
- Please review the other statements to see if your idea is already there. You may search the list of collected statements using the search function below.


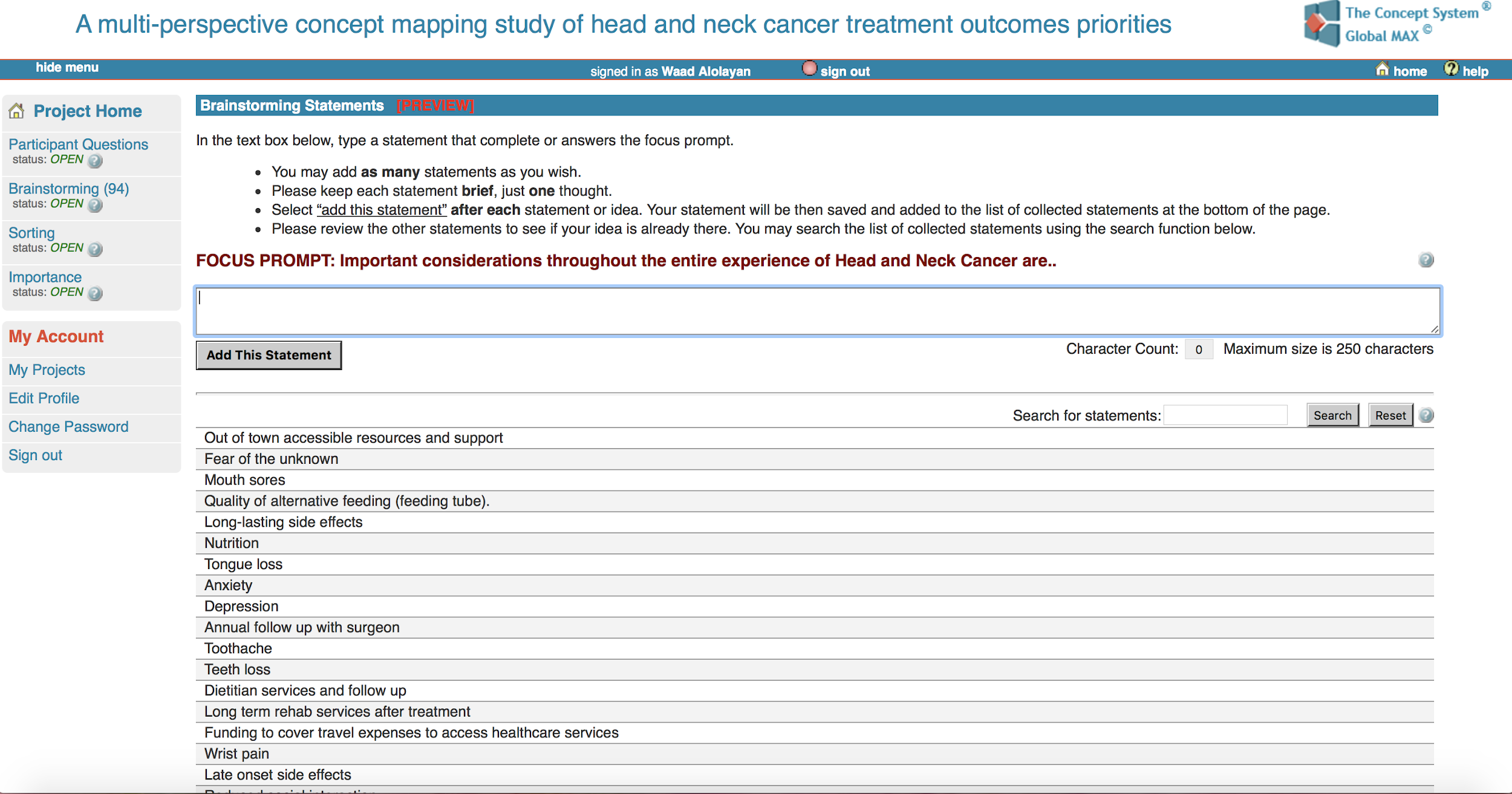

Supplement: S1 Appendix — (DOCX) [file pone.0294712.s001.docx]
