# Supplementary material for: Head and neck cancer treatment outcome priorities: A multi-perspective concept mapping study
Source: PLoS One. 2023 Nov 30;18(11):e0294712. doi: 10.1371/journal.pone.0294712 (PMC10688684; doi:10.1371/journal.pone.0294712)

**S2 Appendix**

**Sorting Activity Instructions**

In this phase, you will categorize the statements according to ***your view of how similar*** *in meaning they are to one another.*

You will sort each statement into piles in a way that makes sense to you.

1. First, read through the statement in the unsorted statements column to the left.
2. Next, **sort** each statement into a pile you create (**by dragging each statement to the right blank window**). Group the statements for how similar in meaning or theme they are to one another.
3. Next, give each pile a **name** that describes its theme or content.

- **Do not** create piles according to the priority or importance, such as *“important”.*
- **Do not** create piles such as *“other”* that contain dissimilar statements together.
- **Put a statement alone in its own pile if it is unrelated to all other statements.**
- Make sure **every** statement is put somewhere.
- **Do not** leave any statements in the unsorted statements column.
- There is **no right or wrong** way to group the statements.
- People vary in how many piles they create.


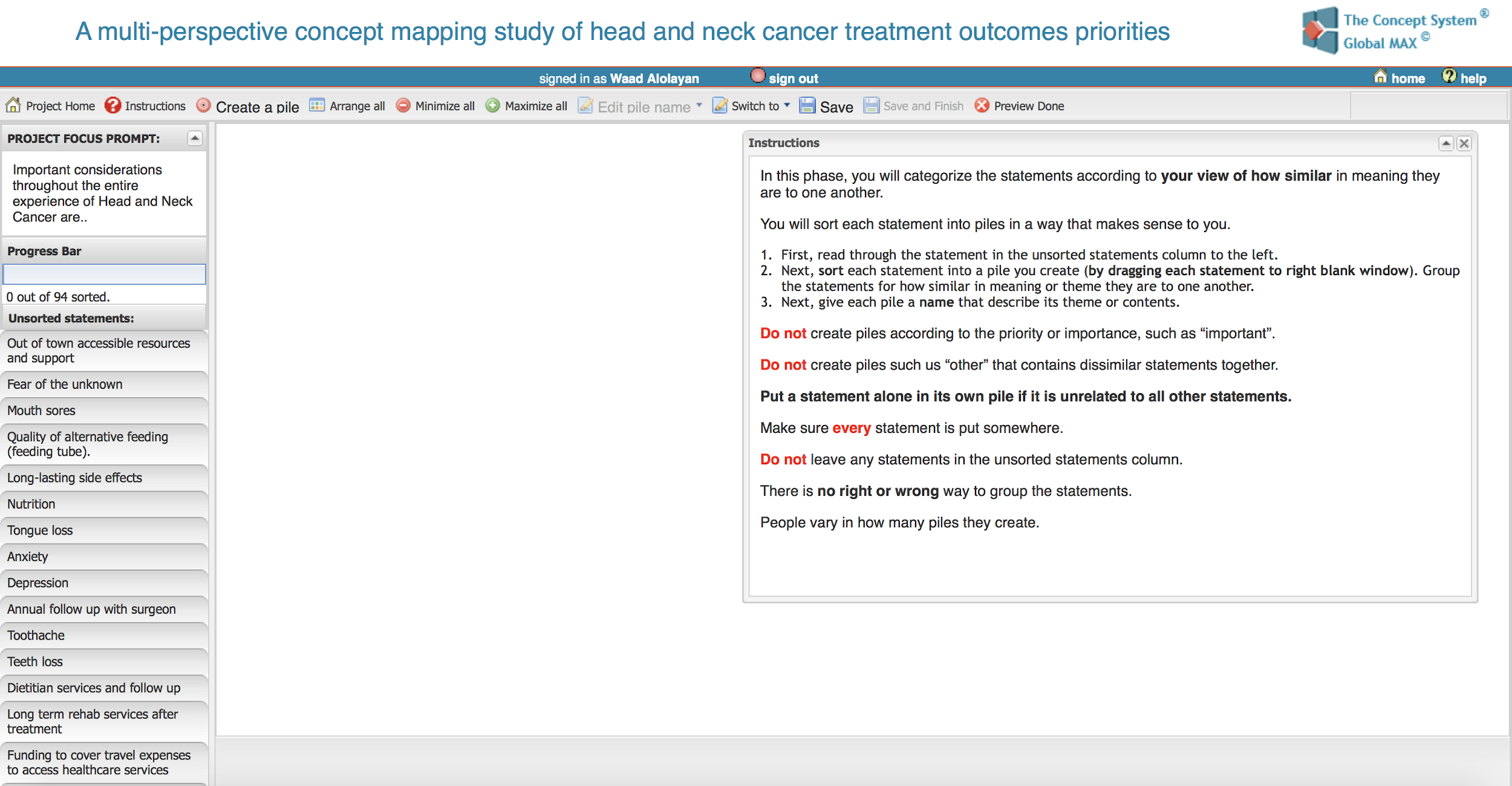

Supplement: S2 Appendix — (DOCX) [file pone.0294712.s002.docx]
