# Supplementary material for: Head and neck cancer treatment outcome priorities: A multi-perspective concept mapping study
Source: PLoS One. 2023 Nov 30;18(11):e0294712. doi: 10.1371/journal.pone.0294712 (PMC10688684; doi:10.1371/journal.pone.0294712)

**S3 Appendix**

**Rating Activity Instructions**

**Instructions:**

On a scale of 1 to 5, rate each statement based on it is **importance/priority** to you.

1= Not important at all.

2= Not very important.

3= Somewhat important

4= Very important.

5= Extremely important.


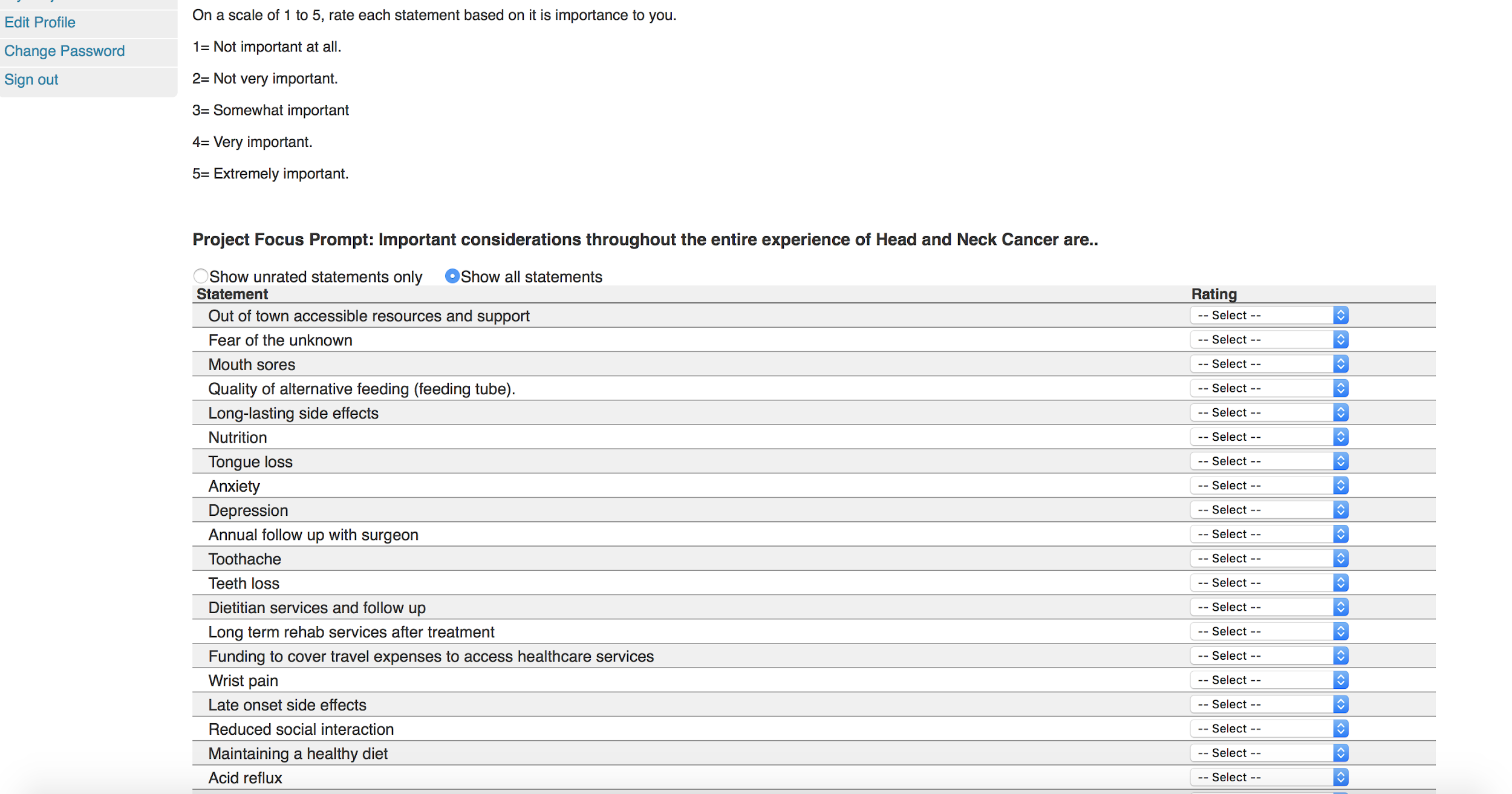

Supplement: S3 Appendix — (DOCX) [file pone.0294712.s003.docx]
