# Supplementary material for: Head and neck cancer treatment outcome priorities: A multi-perspective concept mapping study
Source: PLoS One. 2023 Nov 30;18(11):e0294712. doi: 10.1371/journal.pone.0294712 (PMC10688684; doi:10.1371/journal.pone.0294712)
Supplement: S4 Appendix — (DOCX) [file pone.0294712.s004.docx]

**S4 Appendix**

**List of Final Statements Included in Phase Two**

1. Out of town accessible resources and support
2. Fear of the unknown
3. Mouth sores
4. Quality of alternative feeding (feeding tube).
5. Long-lasting side effects
6. Nutrition
7. Tongue loss
8. Anxiety
9. Depression
10. Annual follow up with surgeon
11. Toothache
12. Teeth loss
13. Dietitian services and follow up
14. Long term rehab services after treatment
15. Funding to cover travel expenses to access healthcare services
16. Wrist pain
17. Late onset side effects
18. Reduced social interaction
19. Maintaining a healthy diet
20. Acid reflux
21. Scheduling/booking follow-up appointments with healthcare providers (e.g., specialists)
22. Changes in voice
23. Access to treatment services in home to prevent unnecessary hospital visits and infection exposure
24. Headache
25. Inability to return to the workforce
26. Tongue pain
27. Dental service providers specialized/experienced in head and neck cancer
28. Surgical scars
29. Accessibility to healthcare services for out of town patients
30. Promptness of diagnosis
31. Ability to communicate and speak intelligibly
32. Costs of parking permits
33. Aspiration
34. Dental follow up after treatment
35. Physical limitation
36. Clear detailed upfront information of the case and treatment plan
37. Stress
38. Prompt Speech and Language Pathology consultation, services and follow up
39. Having mentors who share the same experience
40. Swallowing difficulty
41. Psychological support and recommendations before treatment
42. Education on how to manage and cope with long-term side effects
43. Shortness of breath
44. Post-surgery infections
45. Accessibility of counseling services in cancer treatment centres
46. Understanding statistics and current outcomes
47. Growing beard
48. Dry mouth
49. Ability to taste food
50. Survival
51. Identification of support groups and resources specific to head and neck cancer
52. Hearing loss
53. family/caregiver/loved one/social support
54. Insomnia
55. Support through treatment and recovery
56. Support and guidance after treatment
57. Tracking patients’ progress throughout treatment and post treatment
58. Coverage for allied health and clinical services i.e: dental, rehab
59. Knowledgeable and experienced healthcare providers in head and neck cancer
60. Lymphedema (Lymph fluid retention causing limb swelling where lymph nodes are affected by cancer treatment)
61. Sticky saliva and phlegm
62. Social isolation
63. Keeping the family informed
64. Oesteoradionecrosis (bone death secondary to radiation therapy)
65. Changes in bone density
66. Weight loss.
67. Hair-free skin grafts
68. Graft resorption
69. Pretreatment education on post treatment outcomes
70. Tongue pain
71. Stiff neck
72. Saving saliva glands
73. Promptness of treatment
74. Having an advocate health care provider/nurse/clinician
75. Information on the available treatment options and new advancements
76. Postherpetic Neuralgia (complication of an infection causing burning sensation to skin and fibers)
77. Fear of recurrence
78. Information on long-term side effects and the possible traumatic experience
79. Ultra sensitivity to smell
80. Involve patient in decision making
81. Fibrosis
82. Cracked lips
83. Sore shoulder
84. Stomach sickness
85. Freedom to choosing the medical care providers
86. Accommodation for out of town patients
87. Engaged healthcare providers with compassionate care
88. Fatigue
89. Continue to follow up after 5 years
90. Social worker/counselling support (individual or group)
91. Proper fit of dentures after surgery
92. Communication with healthcare providers
93. Metallic taste in mouth
94. Providing an accessible resource/tool for information and common questions
