# Supplementary material for: Head and neck cancer treatment outcome priorities: A multi-perspective concept mapping study
Source: PLoS One. 2023 Nov 30;18(11):e0294712. doi: 10.1371/journal.pone.0294712 (PMC10688684; doi:10.1371/journal.pone.0294712)
Supplement: S5 Appendix — (DOCX) [file pone.0294712.s005.docx]

**S5 Appendix**

**List of Clusters and Statements (Ascending Rating Value)**

| ***Cluster*** |  | ***Statement*** | ***Average Rating*** | |  |  |
| --- | --- | --- | --- | --- | --- | --- |
| **1. Monetary Support** | | | **3.47** |  |  |  |
|  | 58 | Coverage for allied health and clinical services i.e: dental, rehab | 4.14 |  |  |  |
|  | 29 | Accessibility to healthcare services for out of town patients | 3.82 |  |  |  |
|  | 86 | Accommodation for out of town patients | 3.59 |  |  |  |
|  | 15 | Funding to cover travel expenses to access healthcare services | 3.45 |  |  |  |
|  | 1 | Out of town accessible resources and support | 3.3 |  |  |  |
|  | 32 | Costs of parking permits | 2.5 |  |  |  |
| **Count** | **Std. Dev.** | **Variance** | **Min** | **Max** | **Average** | **Median** |
| **6** | **0.51** | **0.26** | **2.5** | **4.14** | **3.47** | **3.52** |
| **2. Continuing Care** | | | **4.08** |  |  |  |
|  | 27 | Dental service providers specialized/experienced in head and neck cancer | 4.57 |  |  |  |
|  | 34 | Dental follow up after treatment | 4.41 |  |  |  |
|  | 89 | Continue to follow up after 5 years | 4.36 |  |  |  |
|  | 10 | Annual follow up with surgeon | 4.3 |  |  |  |
|  | 45 | Accessibility of counseling services in cancer treatment centres | 4.05 |  |  |  |
|  | 38 | Prompt Speech and Language Pathology consultation, services and follow up | 4.05 |  |  |  |
|  | 13 | Dietitian services and follow up | 4.04 |  |  |  |
|  | 14 | Long term rehab services after treatment | 4.04 |  |  |  |
|  | 21 | Scheduling/booking follow-up appointments with healthcare providers (e.g., specialists) | 3.87 |  |  |  |
|  | 23 | Access to treatment services in home to prevent unnecessary hospital visits and infection exposure | 3.09 |  |  |  |
| **Count** | **Std. Dev.** | **Variance** | **Min** | **Max** | **Average** | **Median** |
| **10** | **0.39** | **0.15** | **3.09** | **4.57** | **4.08** | **4.05** |
| **3. Psychosocial Concerns** | | | **3.87** |  |  |  |
|  | 77 | Fear of recurrence | 4.18 |  |  |  |
|  | 62 | Social isolation | 4.14 |  |  |  |
|  | 37 | Stress | 4.05 |  |  |  |
|  | 2 | Fear of the unknown | 3.91 |  |  |  |
|  | 8 | Anxiety | 3.91 |  |  |  |
|  | 9 | Depression | 3.87 |  |  |  |
|  | 25 | Inability to return to the workforce | 3.52 |  |  |  |
|  | 18 | Reduced social interaction | 3.39 |  |  |  |
| **Count** | **Std. Dev.** | **Variance** | **Min** | **Max** | **Average** | **Median** |
| **8** | **0.26** | **0.07** | **3.39** | **4.18** | **3.87** | **3.91** |
| **4. Nutrition** | |  | **4.15** |  |  |  |
|  | 50 | Survival | 4.68 |  |  |  |
|  | 6 | Nutrition | 4.35 |  |  |  |
|  | 19 | Maintaining a healthy diet | 4.22 |  |  |  |
|  | 4 | Quality of alternative feeding (feeding tube). | 4.17 |  |  |  |
|  | 91 | Proper fit of dentures after surgery | 3.32 |  |  |  |
| **Count** | **Std. Dev.** | **Variance** | **Min** | **Max** | **Average** | **Median** |
| **5** | **0.45** | **0.2** | **3.32** | **4.68** | **4.15** | **4.22** |
| **5. Education** | |  | **4.28** |  |  |  |
|  | 30 | Promptness of diagnosis | 4.86 |  |  |  |
|  | 36 | Clear detailed upfront information of the case and treatment plan | 4.64 |  |  |  |
|  | 75 | Information on the available treatment options and new advancements | 4.55 |  |  |  |
|  | 78 | Information on long-term side effects and the possible traumatic experience | 4.41 |  |  |  |
|  | 94 | Providing an accessible resource/tool for information and common questions | 4.27 |  |  |  |
|  | 42 | Education on how to manage and cope with long-term side effects | 4.23 |  |  |  |
|  | 69 | Pretreatment education on post treatment outcomes | 3.86 |  |  |  |
|  | 46 | Understanding statistics and current outcomes | 3.45 |  |  |  |
| **Count** | **Std. Dev.** | **Variance** | **Min** | **Max** | **Average** | **Median** |
| **8** | **0.42** | **0.18** | **3.45** | **4.86** | **4.28** | **4.34** |
| **6. Person-Centred Care** | | | **4.2** |  |  |  |
|  | 73 | Promptness of treatment | 4.91 |  |  |  |
|  | 59 | Knowledgeable and experienced healthcare providers in head and neck cancer | 4.82 |  |  |  |
|  | 55 | Support through treatment and recovery | 4.59 |  |  |  |
|  | 87 | Engaged healthcare providers with compassionate care | 4.59 |  |  |  |
|  | 80 | Involve patient in decision making | 4.36 |  |  |  |
|  | 56 | Support and guidance after treatment | 4.32 |  |  |  |
|  | 53 | family/caregiver/loved one/social support | 4.27 |  |  |  |
|  | 92 | Communication with healthcare providers | 4.27 |  |  |  |
|  | 57 | Tracking patientsâ€™ progress throughout treatment and post treatment | 4.18 |  |  |  |
|  | 74 | Having an advocate health care provider/nurse/clinician | 4.05 |  |  |  |
|  | 90 | Social worker/counselling support (individual or group) | 4 |  |  |  |
|  | 41 | Psychological support and recommendations before treatment | 4 |  |  |  |
|  | 63 | Keeping the family informed | 4 |  |  |  |
|  | 85 | Freedom to choosing the medical care providers | 3.73 |  |  |  |
|  | 51 | Identification of support groups and resources specific to head and neck cancer | 3.68 |  |  |  |
|  | 39 | Having mentors who share the same experience | 3.41 |  |  |  |
| **Count** | **Std. Dev.** | **Variance** | **Min** | **Max** | **Average** | **Median** |
| **16** | **0.4** | **0.16** | **3.41** | **4.91** | **4.2** | **4.23** |
| **7. Treatment Side Effects** | | | **3.43** |  |  |  |
|  | 40 | Swallowing difficulty | 4.36 |  |  |  |
|  | 72 | Saving saliva glands | 4.32 |  |  |  |
|  | 64 | Oesteoradionecrosis (bone death secondary to radiation therapy) | 4.23 |  |  |  |
|  | 44 | Post-surgery infections | 4.18 |  |  |  |
|  | 88 | Fatigue | 4.05 |  |  |  |
|  | 48 | Dry mouth | 4 |  |  |  |
|  | 35 | Physical limitation | 3.95 |  |  |  |
|  | 49 | Ability to taste food | 3.91 |  |  |  |
|  | 61 | Sticky saliva and phlegm | 3.86 |  |  |  |
|  | 54 | Insomnia | 3.82 |  |  |  |
|  | 33 | Aspiration | 3.82 |  |  |  |
|  | 65 | Changes in bone density | 3.77 |  |  |  |
|  | 52 | Hearing loss | 3.77 |  |  |  |
|  | 3 | Mouth sores | 3.65 |  |  |  |
|  | 66 | Weight loss. | 3.64 |  |  |  |
|  | 11 | Toothache | 3.59 |  |  |  |
|  | 60 | Lymphedema (Lymph fluid retention causing limb swelling where lymph nodes are affected by cancer treatment) | 3.5 |  |  |  |
|  | 70 | Tongue pain | 3.5 |  |  |  |
|  | 84 | Stomach sickness | 3.45 |  |  |  |
|  | 76 | Postherpetic Neuralgia (complication of an infection causing burning sensation to skin and fibers) | 3.41 |  |  |  |
|  | 71 | Stiff neck | 3.36 |  |  |  |
|  | 43 | Shortness of breath | 3.36 |  |  |  |
|  | 26 | Tongue pain | 3.35 |  |  |  |
|  | 81 | Fibrosis | 3.18 |  |  |  |
|  | 79 | Ultra sensitivity to smell | 3.18 |  |  |  |
|  | 20 | Acid reflux | 3.05 |  |  |  |
|  | 93 | Metallic taste in mouth | 3 |  |  |  |
|  | 24 | Headache | 2.96 |  |  |  |
|  | 83 | Sore shoulder | 2.86 |  |  |  |
|  | 68 | Graft resorption | 2.77 |  |  |  |
|  | 82 | Cracked lips | 2.55 |  |  |  |
|  | 67 | Hair-free skin grafts | 2.36 |  |  |  |
|  | 16 | Wrist pain | 2.13 |  |  |  |
|  | 47 | Growing beard | 1.68 |  |  |  |
| **Count** | **Std. Dev.** | **Variance** | **Min** | **Max** | **Average** | **Median** |
| **34** | **0.62** | **0.38** | **1.68** | **4.36** | **3.43** | **3.5** |
| **8. Lifelong Challenges** | | | **3.74** |  |  |  |
|  | 7 | Tongue loss | 4.36 |  |  |  |
|  | 31 | Ability to communicate and speak intelligibly | 4.32 |  |  |  |
|  | 5 | Long-lasting side effects | 4.17 |  |  |  |
|  | 12 | Teeth loss | 4.04 |  |  |  |
|  | 17 | Late onset side effects | 3.78 |  |  |  |
|  | 22 | Changes in voice | 2.91 |  |  |  |
|  | 28 | Surgical scars | 2.59 |  |  |  |
| **Count** | **Std. Dev.** | **Variance** | **Min** | **Max** | **Average** | **Median** |
| **7** | **0.66** | **0.43** | **2.59** | **4.36** | **3.74** | **4.04** |
