# Supplementary material for: Head and neck cancer treatment outcome priorities: A multi-perspective concept mapping study
Source: PLoS One. 2023 Nov 30;18(11):e0294712. doi: 10.1371/journal.pone.0294712 (PMC10688684; doi:10.1371/journal.pone.0294712)
Supplement: S6 Appendix — (DOCX) [file pone.0294712.s006.docx]

**S6 Appendix**

**Analysis Process in Group Concept Mapping**

1. Core mapping (sorting) analysis: analysis of the sorting data to create the visual point maps and cluster maps. It is conducted through three steps:
   1. Similarity matrix: the similarity matrix covers all sorting data and information in a project and it shows the number of participants who sorted each pair of statements together on a table. For one participant, there are as many columns and rows in the table (the individual matrix) as the statements that were sorted, a value of either 1 or 0 is plotted across the table. A ‘1’ value indicates that these statements were grouped together and a ‘0’ value indicates that the statements were not grouped together by the participant. Next, a similarity matrix is created as cells in all individual matrices are summed to include all participants' sorting. The values are more than 0 and 1in this matrix (table) and are a resembling of the number of participants who sorted the statements together. Hence, the high values indicate a high number of participants who sorted these statements in the same group.
   2. Nonmetric multidimensional scaling of the similarity matrix: This step of analysis where the qualitative data (statements) are converted to quantitative data (x,y) points. It is illustrated as the point map, it sites each statement as a distinct point on a two dimensional (x,y) map to depict the relationship among statements in terms of proximity.
   3. Hierarchical cluster analysis of the multidimensional scaling coordinates: groups (x,y) points into clusters that indicate similar concepts. It begins with each statement as a cluster, and the algorithms then combine two clusters, and continue to combine until all statements are under one cluster.
2. Bridging-Anchoring analysis: to show the anchoring and bridging statements and/or clusters in a certain area on the maps. The bridging value is the degree to which a statement was sorted within a cluster or with other statements in different clusters by participants, in other words, it is an index of how cohesive are the statements within a cluster, and it’s a measure with a value from (0-1). The closer the value to 0, indicates that the statement is related to other nearby statements “anchor”. The higher the value, indicates that the statement is related to other statements elsewhere on the map “bridging” (Kane & Trochim, 2007).
3. Cluster label analysis: to choose the best representative label of each cluster, based on the labels given by participants. Although the software now suggests representative labels based on participants’ input, the researcher can also add different labels without using the participants’ labels if seen more representative of the cluster's content. The researcher meanwhile needs to have a rich comprehension of the relationship of statements within the cluster (Kane & Trochim, 2007).
4. Determining the final number of clusters using the hierarchical cluster analysis. Number of clusters on the cluster map can be determined by the researcher and team judgement. The bridging value of each cluster can also be used for this purpose (Kane & Trochim, 2007). This complex process depends on the researcher’s knowledge of the methodology and topic studied to arrive at the most useful number of clusters that reflects sufficient contextual details across clusters and merge the ones that belong together in sense. To pursue that, it is recommended to choose a maximum and minimum number of clusters that are efficient in reflecting the desired context, and starts examining the hierarchical analysis of the merging clusters within this band to arrive at the final number (Kane & Trochim, 2007).
5. Rating analysis: using the rating data to create the rating maps; cluster rating maps, pattern matches, and go zones. Rating analysis is based on averaging a single statement rating across participants.


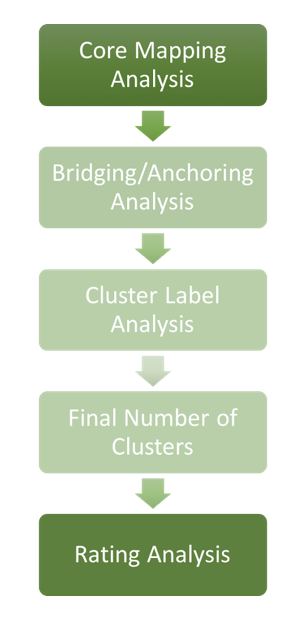


Analysis Process. This is a flowchart of the analysis process in GCM
