# Supplementary material for: Head and neck cancer treatment outcome priorities: A multi-perspective concept mapping study
Source: PLoS One. 2023 Nov 30;18(11):e0294712. doi: 10.1371/journal.pone.0294712 (PMC10688684; doi:10.1371/journal.pone.0294712)

**S7 Appendix**

**Consent**

You have been asked to participate in a web-based project. Your participation is voluntary.

You may be asked to offer your input in a variety of ways:

by providing non-identifying information about yourself.

by providing your ideas

rating the ideas or sorting them into groups of similar themes

You may participate in the entire project or in any one aspect of the project. Your input in this project is confidential.


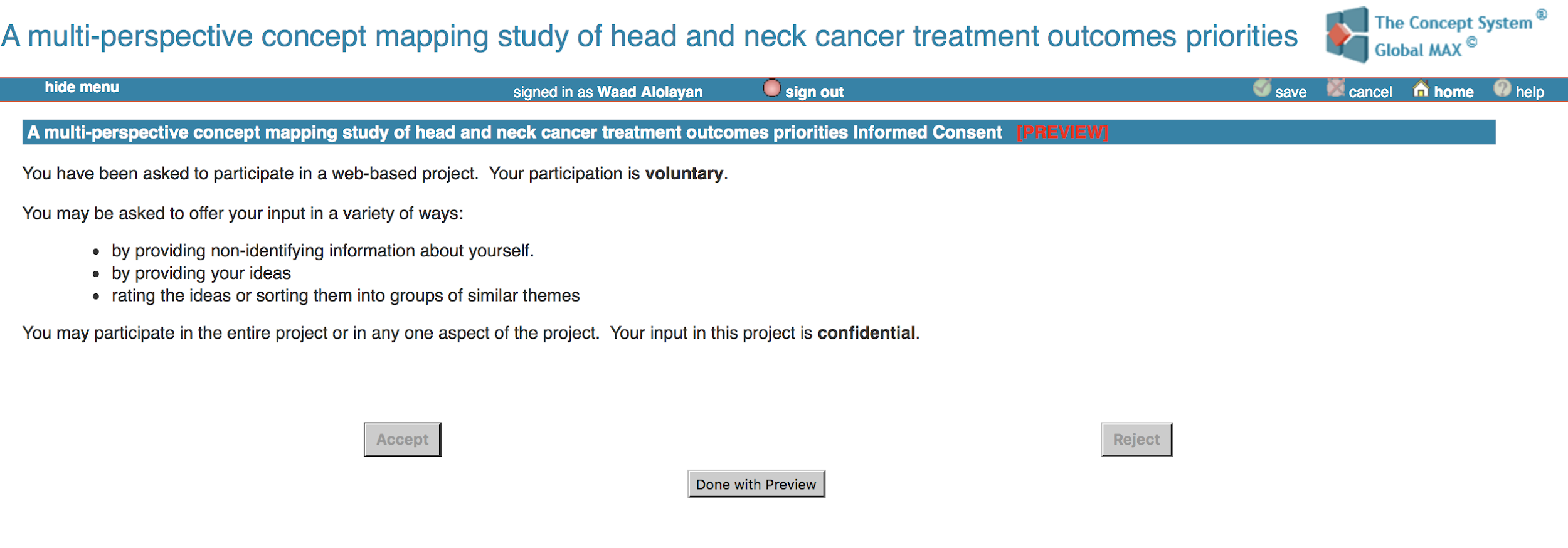

Supplement: S7 Appendix — (DOCX) [file pone.0294712.s007.docx]
