# Supplementary material for: Head and neck cancer treatment outcome priorities: A multi-perspective concept mapping study
Source: PLoS One. 2023 Nov 30;18(11):e0294712. doi: 10.1371/journal.pone.0294712 (PMC10688684; doi:10.1371/journal.pone.0294712)
Supplement: S9 Appendix — (PDF) [file pone.0294712.s009.pdf]

1. Surgeon and his team that we had explained all the possible scenarios and indeed the pt had most of them, because everything had been explained the pt was less anxious and caregiver could remind the pt of what to expect. Education works.
2. Having compassionate care from engaged health care professionals is extremely important.
3. Promptness of diagnosis and treatment is equally important.
4. I would like to see more emphasis on preventative measures with increased education for dentists and dental hygienists, specifically the signs of chronic, long-term oral cavity infections.
5. I would like to see the establishment of a dental diagnostic unit with access to CT scanning where patients with inexplicable jaw pain, chronic oral inflammation, chronically enlarged level 2 lymph nodes or spreading inflammation could be referred.
6. swallowing very difficult. Aspiration big risk.
7. Not told long term effects of radiation. e.g. bone density, teeth falling out.
8. Can't afford all dental work.Should be covered by medicare.
9. Dental options lacking
10. Thankful you're alive rang hollow.
11. People said you can be thankful you're alive. This is so true. But it sometimes rang hollow. The lasting effects and just wanting to feel healed and going back to being normal was what I really wanted.
12. For the first 2 or 3 months after treatments, looking in the mirror, seeing surgery neck and throat scars reminded me that I had cancer. I cried when I touched my neck. This visual reminded me of cancer. It was depressing.
13. Radiation has effected my mouth. Constantly have mouth sores. Easy to bite tongue, lips and sides of mouth.
14. Radiation caused loss of hearing. Experienced this effect a few years after end of treatments.
15. Changes to my voice have been significant to my life. As a teacher and public speaker voice change has been perhaps hardest adjustment. It's like losing full function of a limb.
16. Excellent counselling support post radiation at Med Hat cancer clinic
17. The counselling services moved downtown off Tom Baker site. My illness and facility inconvenience prohibited me from accessing counselling while undergoing treatments.
18. Unexpected ultra sensitivity to smells. Strong odours overwhelming causing nausea. Couldn't stay at Foothills hostel because of construction odours. Friends fortunately supplied accommodation.
19. My Med Hat ent doctor quickly diagnosed, operated on cancer. Calgary patients I met didn't receive the same expedient attention. Radiation treatments in Calgary so travels to city from Med Hat soon became exhausting. Incurred expenses.
20. Speech therapy works! Practice, practice, practice :)
21. Try to be positive, it is no fun but there is a light at the end of the tunnel and you will get there.
22. support group specific to head and neck cancer
23. It would be appreciated if there was funding or assistance for post surgery and treatment for dental issues specific to Head and Neck Cancer patients
24. Dental options were lacking 19 years ago that now make dental appearance and health a challenge
25. 19 years post 1st surgery. Quality of life is very good. Only 1 reasonably significant (to me) issue at present, that dental did not seem to be much of a concern at the time of my surgery and radiation.
26. Support by loved ones

27. Recent compelling articles compare state of post cancer treatment and recovery as PTSD...life altering...
28. Be prepared for problems with ears and eyes from radiation scatter...they don't tell you that either!!!
29. Be prepared for "Chemo and Radiation Brain"...they don't tell you that ahead of time!!!
30. Access to the Cross is abysmal...lineups to park...aren't patients taxed enough financially without having to pay $2.25/half hour. What happens if clinic is running behind, you go over and end up with a parking ticket...are you kidding me???
31. Stage 4 missed by 3 professionals...GP, ENT and Emerg...last emergency finally sent me to UofA Head n Neck...
32. Time from diagnosis to actual commencement of treatment, much to long...hurry up and wait...
33. Quality of life post treatment is a rare commodity. No where near enough attention paid to long term issues.
34. Nurse practitioner positions should quadruple...an integral extension of overworked Oncologists...
35. The surgeon, who didn't end up doing surgery, followed up better than anyone at the cancer clinic??
36. Have volunteered to mentor on several occasions and have never been contacted.
37. Support groups along with counselling should extend long beyond treatment.
38. Surgery is not absolutely necessary...can now be done robotically...get opinions before consenting to invasive procedures.
39. Many patients are alone and need some form of support.
40. One on one councillor (Social Worker) discussions should be mandatory...before, during and post treatment...for as long as is necessary.
41. Cancer clinics in Alberta working at 250%...where are the resources... Oncologists don't have the time to do their jobs anywhere near to the capacity they wish to.
42. Three months of rehab post treatment, aren't anywhere near enough. Issues have cropped up six years after initial treatment.
43. When cancer treatment stops, so seems after treatment care. Chemo and Radiation...the gifts that keep on giving for years. No patient follow-up. Family docs & Specialists left to pick-up pieces. They don't have the cancer knowledge needed.
44. Exercise before and after saved me physically and mentally.
45. Post treatment support. You ring the bell (which is exhilarating) and then you go off to the unknown
46. Supporting patients with tracking information
47. The age of the patient.
48. Making connections with patients and their families.
49. We need to change our focus, treat the patient first, disease second. Bedside manners can be improved
50. Avoid sugars!! Eat green! Stay positive!
51. Waiting times are critical! The sooner you get to see your specialist the better your survival.
52. I was missed diagnosed that my tonsil had tonsil stones on it but instead it was cancer and piroity was put on two small lumps on my thyroid instead, which was not cancer. I was overlooked by my GP and radiologist. I should have been sent to a spec
53. I feel there needs to be some change in the liquid food that is being fed through the feeding tube. Cancer loves sugar and the first ingredient is sugar. Also not everyone can eat this. Eating heathy is extremely important.

54. Care after surgery,nurses and hospital caregivers, should have compassion fthat the patient there is many tubes you, can't speak or move easily. Therefore needing help. Tracheotomy is very scary when if gets clogged
55. it's great to be alive!
56. the ACE (Alberta Cancer Exercise) program was great as a follow-up to cancer and offered locally (as in other than Calgary/Edmonton)
57. My local ENT was stumped when I went to him for diagnosis (tongue cancer). When sent to Calgary they knew before biopsy what it was. More education?
58. No communication between care and surgery in Calgary and then going home to Lethbridge. Had to find out about free cancer physiotherapy from a friend. More communication needs to happen between the Tom Baker & Jack Ady (or where you live) Centres
59. prior health and the importance of preparing the body for surgery and treatment.
60. running around for diagnostics. live in lethbridge, scanned in medicine hat, operated in calgary, post surgery followup in calgary...all travel at personal expense and leave of absence financial loss
61. Diagnosis - took 3 months. Blood tests, 2 ultrasounds. Family Doctor didn't listen as I complained about pain with eating, swallowing taking meds. Had to go to emergency to get to specialist who had to perform 2 biopsies. He didn't mess around.
62. I highly recommend yoga. I started a few months after my treatment ended and it has been transformative; both physically and mentally. I love hot vinyassa flow, but there are different yoga styles to suit all tastes and abilities.
63. Managing information (what can I expect?), pain (unexpected and severe) and weight loss (one third of body weight in six weeks).
64. Having the right medical team - knowing that it is your right to choose your medical providers
65. Knowing that cancer and chemo impact bone density
66. Oral support information
67. Speed of getting the treatment
68. Medical support, family support, Knowledge
69. Patience, following treatment
70. Support; proper information and strength.
71. How do I deal with the effects of radiation afterwards? I had to figure it out on my own. I was relieved for followup checkups at the Cross.They checked for cancer.I wanted something to help deal with the impact of radiation therapy.
72. My family doctor wa unable to get me in to someone who could actually identify what kind of cancer I had. Getting in to a dermatologist he told me not to worry it was basal cell, a lazy cancer. The next doctor told me my cancer was squamous cell.
73. education to family doctors - mine had no idea what was going on - once in the hospital many other patients had exact symptoms as me! It could have been caught sooner
74. co-workers became used to my speech in time and with that came more confidence and more improvements
75. Speech - it improves by doing! listen to yourself and find ways to improve along with therapy. Mine improved drastically once back at work with the need to communicate.
76. Phlem/sticky saliva - an oral vacuum like the dentists use (cheaper version) I think would be a huge asset
77. stay socialable, accept visitors
78. Use imagination when it comes to eating, things you disliked before may become a favorite just because it is easy to get down and/or taste buds change
79. My entire medical team was awesome, from pre surgery through to end of chemo,radiation and physio. Use every available option accessible

80. Throw vanity out the window, be comfortable and be selfish while you recouperate
81. Everyone is different, recuperation times and quality of life issues will be effected differently for each person. Do what you have to for yourself, not based on the outcome of others.
82. Exercise and eat well otherwise the fatigue will get you.
83. You become a hermit over a period of time as your health deteriorates.
84. Hungry always hungry. Feeding tube gets kind of boring and tedious after a while, I do not eat enough even though I know I have to. Gnaw at the sides of my mouth when sleeping and dream of all sorts of fattening foods. Pizza, burgers, fries etc.
85. Constantly dealing with phlegm
86. Bad metallic taste in mouth
87. Social interaction dramatically reduced
88. Reduced ability to communicate
89. Overall loss of quality of life
90. Food as a part of life
91. Active, direct and consistent caregiver/spouse support throughout diagnosis and treatment of loved one....compassion fatigue is overwhelming
92. Provide awareness for cancer treatment teams of EMS Mobile Integrated Health, paramedics which can provide in-home hydration, pain control, lab draws, POCT, blood administration...preventing unnecessary hospital visits and infection exposure
93. Physical rehab support needs to be provided and funded for at least two years...radiation fibrosis, lymphedema, surgical rehab is critical in providing physical support for patients to recover and progress towards a new normal
94. Develop and implement patient navigator positions to follow-up, guide and support patients in post treatment journey...a huge gap exists in support once patients are released from treatment, despite ongoing affects/effects; both physically & mentally
95. GP's in many cases do not know the issues - this is who you see after the clinic kicks you out.
96. Chest pains caused by swallowing issues and dry mouth
97. Dental - what dentists outside of the hospital understand and especially knowledgeable dental hygenists
98. Knowledge - be your own advocate
99. Saving saliva glands
100. Meeting with Dietitians very important.
101. Swallowing and speech specialists very important.
102. Glaxal lotion very important
103. Don't change the chemo rooms it's good to have others around.
104. Having a nurse to be your health advocate would have been excellent.
105. Keep your life as normal as you can.Keep routines as best you can. Keep trying to eat. I went to support group during radiation 'cause I want to know what's next.
106. Not enough information is given about long term side effects:dental issues,breathing problems swallowing problems,neck mobility.
107. Be up front and totally open and honest with patient (and caregiver)
108. Attitude. It is very important to have and foster a positive attitude throughout the process and beyond.
109. Quality of life post treatment.
110. 13 years post surgery and treatment, just continuing to have faith in God. He will not leave or forsake those that are His.
111. Not enough information is given about long term side effects: dental issues, breathing problems, swallowing problems, neck mobility, restriction of shoulder movement. There needs to be an accessible list of dentists that are familiar with

survivors of head and neck cancer so that dental problems can be dealt with in a competent manner with the least amount of harm done.

112. out of town patients need to know what resources are available such as housing locations, support groups.

113. keep your life as normal as you can. Keep routines as best as you can. Modify them like exercise, BUT still do them. Eat -even if it is just a bit -keep trying to eat. I went to the support group during my radiation 'cause I want to know what's next.

114. Having a nurse assigned to be your health advocate would have been excellent.

115. Introduction to others going through the same experience is critical.

116. Remember it is not your fault you got cancer. In my case I was a never smoker, not overweight, ate all the right things, was healthy and active, and only 42. It's just bad luck.

117. Not enough information is given about long term side effects: dental issues, breathing problems, swallowing problems, neck mobility, restriction of shoulder movement.

118. There needs to be an accessible list of dentists that are familiar with survivors of head and neck cancer so that dental problems can be dealt with in a competent manner with the least amount of harm done.

119. Long term survivors still need regular appointments with ENTs to make sure everything is fine, any re occurrence can be caught early, and to give a feeling of control .

120. social support and support group network connecting with patients through recovery process would have a positive impact.

121. social support after surgery and during treatment

122. provide information on and while staying in ICU after surgery.

123. the appearance and poor speech intelligibility to apply for a job after treatment.

124. not being able to return to workforce after treatment due to physical limitation.

125. reconstruction of sensation areas to maintain full function.

126. follow up for some sort of dentition to maintain smile.

127. provide information, support and continuity of care on how to deal with cancer after surgery.

128. psychological support and recommendations to be available after surgery for patients.

129. the physical treatment is not sufficient, and the concerns are long lasting than the surgery room

130. understanding the traumatic nature of treatment outcomes

131. the surgery outcomes were fine, but the psychological impacts were unclear

132. couldn't grow beard "appearance change" after radiation therapy, which changed identity

133. loosing teeth and having dentures fit properly after surgery.

134. the ability to speak intelligibly

135. Make sure you have them zap the hairs off of any skin graft they put in your month, if not it can lead to a pretty hairy experience.

136. RSM have swallowing and speach specialists, ask your doctor to set you up with them.

137. You go through so much from your initial diagnosis through surgery, radiation or chemotherapy, to perhaps infection and a second surgery, through rehab, hyperbaric chamber, new implants, feeding tube 6 month check-ups then hopefully 1 year check-ups.

138. Depression, Anxiety, Stress, fatigue, these are some of the things that you will experience after surgery.

139. I never knew I was going to have radiation until the day I was being released. I was dressed and packed waiting for my wife to pick me up and instead it was an ambulance to take me from the U of A to the Cross. Shocking mis-communication.
140. Other than surgery Be prepared for some things you never expected. Like radiation, hyperbaric chamber and chemotherapy. Or eventual tube feeding. Lots of hours 31 days straight after your surgery. Then if you are lucky enough to qualify for implants.
141. I received Implants on my lower teeth. Nothing but good things to say about Doctor Oswald at RSM and misericordia dental team.
142. Get up and about when you are able after your surgery. Hospitals can be kind of boring but you would be surprised all the stuff you can discover there. I did the halls a lot after my surgery and became very familiar with whats to see and do.
143. If you are on your second surgery and know a lot more try and help the patients on the ward with suggestions and share your first experience with them.
144. After your surgery when you are in lots of pain make sure that your doctor has good drugs for this on his orders for you. I used Morphine, percoset and Dilated worked good. Had every 4 hours. I made sure I was in no pain always.
145. See your Dietitian at U of A for group meetings, I think that they have them every 2nd thursday of each month. I am not sure if someone who has not gone through the surgery yet can attend these or not. I believe that they start around 2:00 PM.
146. Get into your dentist early, to ensure that your dental care is addressed prior to radiation therapy.
147. To think that I brought this upon myself due to smoking. As the saying goes "I would not wish this on my worst enemy" or the saying "you reap what you sew" you think these types of things as you experience this type of surgery.
148. Nutrition, I am fading away over the years and I still wish that I could eat a cheeseburger.
149. The entire team work very well together, I have had nothing but good care from the beginning to where I am now 9 years later where my care is ongoing with my home nutrition feeding team. Surgeons, nurses, dietitians, physio therapists. All of them.
150. Your appearance is different after a while. You will probably look a bit gaunt as you will lose weight over a period of time. 9 years after my 2 major surgeries I have lost A LOT of my body weight.
151. When you ask what the odds are of something happening? and are given 50/50 it's usually the negative 50 that happens, be prepared.
152. Be prepared to have a second surgery. I had radiation and developed a infection about a year after my surgery and ended up in the hosp. for a couple of weeks, after that I was on heavy duty antibiotic for 6 mths then a year after that another surgery
153. Find out as much as you can about the initial surgery, perhaps go to some of the group sessions for patients who have already done the surgery. Or go up to the ward and discuss with current patients (if they approve). See what you are going into.
154. The big plus is that I am still alive after my first surgery 9 years ago, a serious infection due to radiation (my opinion) and a second surgery 6 years ago. I have no complaints as here I am writing this. However I do have thoughts to improve care.
155. Be compassionate with loved ones. We only have to be sick and they have all the worries and the regular living stuff to contend with. We only have to be sick and fight.
156. A lot of information from old procedures and outcomes (horror stories about relatives etc.) that don't pertain because of new procedures and breakthroughs. Warn new ones when searching internet and talking with people.
157. How do I deal with the constant fear that now treated my cancer will some back.

158. Why was I not given all the information regarding side effects from my various stages of treatment from surgery, radiation and chemo.
159. Being diagnosed with cancer how can I connect with others who have gone through treatment to see what lies ahead for me.
160. Don't change the chemo rooms (some want single private spaces), it's important to be with others and see their different struggles. It is support and learning.
161. Glaxal based lotion with vitamin E should be recommended to all high dose radiation patients with burning(would have went nuts without it)
162. Dry mouth was terribly painful but with learning about Zylimelts (sticking on roof of mouth) it got better. Four years after(chemo radiation radical neck dissection), have had 50% improvement. There is hope but new ones need to know this.
163. Lack of family doctors trained in cancer care. Because of my cancer my family doctor doesn’t want to see me. No idea on how to check for reoccurrence or how to deal with the damaging and painful effects from the radiation treatments.
164. Lack of follow up. Once punted out of the hospital you are on your own to try and survive. You are told to come back in 3 months but you can’t book the follow up appointment when you leave. You are totally on your own to try and rebuild your life.
165. The damage radiation causes to healthy bone was not adequately articulated. The new bone that was exposed to radiation broke down within 2.5 years causing extensive damage and infection compounded by re-occurrence of cancer which led to 2nd surgery.
166. Little to no support for patients outside of Edmonton.
167. More emphasis on nutrition and food prep when being sent home. Learning to rely on food that is not processed. Knowing what equipment will help with food preparation - get a good quality blender.
168. Quick effects of radiation. Lost voice. Difficulty swallowing. Mouth/tongue food sensitivity. Loss of energy/tiredness. Stomach sickness, needing intravenous. Long recovery post radiation. Dealing with pain sensitive to T3, morphine
169. The expense of parking at the Cross is large burden on cancer patients that have to be there for countless weeks of treatment
170. Was not told my stage of cancer by any oncologist until coldly stated by chemotherapist. Should have been told about radiation arcing on dental fillings previous to treatment. This caused major pain and damage to teeth for lifetime. Extreme expense.
171. When I was first diagnosed, I asked my Oncologist, "What is the five-year survival rate for my cancer?" He said, "85-90%". At that point, my dread disappeared and I decided, I'm not going to die so let's concentrate on fighting this and getter better.
172. No support (Edmonton) for out of town elderly man with no family. He was alone taking morphine for pain. I feel he slipped through the cracks in our system
173. I lost my ability to taste food after radiation & chemotherapy. I gradually got it back with mindful eating & gastronomy courses. My taste is now better than it was previous to treatments. Things can be done to improve the long term side effects
174. The more information given out during and immediately post treatment on the expected trajectory of longer term side effects and things that can be done to mitigate them.
175. How to avoid getting depressed.
176. Follow-up appointments with the surgeon yearly, even after 15 years is very helpful for putting your mind at ease. Having my throat scoped every year is important to me to catch the cancer early if it returns.
177. After successful treatment, quality of life becomes the paramount issue. Patients need to know what lies on the the road ahead and how to best navigate the road ahead to give them the best quality of life.

178. Each person is different. It's so hard to make blanket statements for each person as to what their considerations will be. After treatments, I got shingles. After, I got Postherpetic Neuralgia. Other patients had other experience.
179. Keeping active, staying positive, relax when your energy depletes, surround yourself with positive people, remove negativity.
180. It is very serious and go into this with an open mind knowing how serious is and your expectations may have underestimated what you will experience. I thought I'd return to work after a couple of months. I was off work 2 years.
181. Weight loss over a period of time. Get fat before your surgery and indulge yourself in all of the delicious foods that you like. Have one really, really good last meal. Before treatments started, I gained 20 lbs. Ate my favorite foods&weight trained
182. I think it is important to treat the whole person, not just the cancer. It can cause other mental and emotional issues such as depression.
183. The lack of upfront information regarding radiation side effects. These were not fully explained. Radiation is the gift that keeps on giving with side effects show up years later. There are many short term and long term side effects from treatments.
184. When first diagnosed fear can set in. It might be helpful to have counselling available and I would even go so far as to say mandated. There were many thoughts that I could not share with family or friends because they could not possibly relate.The lack of upfront information regarding radiation side effects. These were not fully explained. Radiation is the gift that keeps on giving with side effects show up years later.
185. It might be helpful to have a mentor - someone who has gone through the same procedures to learn from, to be able to ask questions, and to offer support.
186. I believe that more individual and concentrated assistance around the swallowing and speaking would have been helpful.
187. I would have liked more up front information about the failure of implants.
188. When first diagnosed fear can set in. It might be helpful to have counselling available and I would even go so far as to say mandated. There were many thoughts that I could not share with family or friends because they could not possibly relate.
189. I think it is important to treat the whole person, not just the cancer.
190. The lack of upfront information regarding radiation side effects. These were not fully explained. Radiation is the gift that keeps on giving with side effects show up years later.
191. Weight loss over a period of time. Get fat before your surgery and indulge yourself in all of the delicious foods that you like. Have one really, really good last meal. Over a period of nine years I have lost about 30% of my body weight.
192. It is very serious and go into this with an open mind knowing how serious is and your expectations may have underestimated what you will experience. Mine did, I thought that I would lick the cancer right away after surgery and this was not the case.
193. Oh to eat solid food again! it's been nine years for me since my first surgery and I would do anything to have a cheeseburger or any food that I constantly dream about every night. I wish that they could grow me a tongue.
194. That after your first surgery you may need to have another surgery due to infection and lose more of your body parts such as your entire jaw, all or some of your teeth your ability to speak properly and slobber or drool on yourself.
195. That you may not ever eat solid food again. That you will lose your tongue. That you will have a feeding tube eventually. That you may get an infection after surgery that can be very serious and may need to be in the hospitalized again.
196. I think that each patient needs to know and decide prior to treatment weather they will have surgery, chemotherapy and radiation, a combination of two of these or all three.

197. Keeping active, staying positive, relax when your energy depletes, surround yourself with positive people, remove negativity on how much pain you will endure, most important is to trust our lord and savior that he still has a plan for you.
198. Each person is different. It's so hard to make blanket statements for each person as to what their considerations will be. In my case, I got thrush and I couldn't eat or drink during the last part of the treatments and when they were over.
199. After successful treatment, quality of life becomes the paramount issue. Patients need to know what lies on the the road ahead and how to best navigate the road ahead to give them the best quality of life.
200. The patients GP should be knowledgeable on what to look for at checkups and post treatments that would be helpful years down the road as a result of having radiation or chemo, etc.
201. Follow-up appointments with the surgeon yearly, even after 15 years is very helpful for putting your mind at ease.
202. Dealing with post treatment pain. Stiff neck, sore shoulder, dry mouth, aching teeth, cracked lips, head aches, insomnia, shortness of breath, acid reflux, aching wrist, tongue pain, conditions H and N cancer patients live with without relief.
203. Social isolation due to speaking and swallowing issues. Without a voice and not being able to swallow ostracizing one from society.
204. The lack of clear upfront information on what post treatment will look like. The nurse navigator vaguely tells you what the surgery will entail but does not prepare you for post treatment life. You are left to fumble along on your on.
205. Emotional support, more upfront information, better understanding of post treatment options
206. The timelines for diagnosis and possible treatments. Patient communications for treatment necessities and forecasted outcomes.
207. Communicating with busy doctors
208. Accepting grief and loss - tools to move forward to understand change and transition
209. Despite the issues life maybe different but can be good after treatment
210. How to avoid getting depressed
211. Understanding the experience of head and neck cancer will be with you for the rest of your life
212. Long term survival
213. Sensible and practical recommendations about returning to a more "normal" life post treatment including work, exercise, activities.
214. Communication
215. Education, information, someone to talk to who understands (best if they have been there a done it)
216. Education of things one can do to help mitigate the serious effects of treatment both short and long term e.g. swallowing, saliva, speech, dental issues, radiation burns, bone weakening, pain, energy, weight loss etc.
217. Clearly identification of support groups and cancer resources e.g. Wellspring
218. Understanding statistics
219. The more information given out during and immediately post treatment on the expected trajectory of longer term side effects and things that can be done to mitigate them
220. Swallowing issues appear to get worse many years after stabilizing. This becomes a serious lifestyle issue.

221. There should be scheduled follow-up with an ENT doctor who understands the long term implications of treatment on swallowing and speech at least once every two years after the five year mark.
222. Need for detailed consult with a speech and swallowing consultant soon after treatment. They can provide appropriate exercises to try to mitigate future decline in swallow ability.
223. Options
224. Survival rates
225. Not knowing the questions that should be asked, we should be told of the worst possible results from radiation, etc, it would be helpful in making decisions regarding quality of life issues
226. Make more after-treatment considerations, info & care plans available. Care Info for radiated tissue, scar tissue. Information on post treatment issues should be more available, easy to find.
227. Current statistics and outcomes needed to make informed choice on treatment options.
228. A "new patient starter kit" would be welcomed. Notepad & pen for questions, list of FAQ, list of online resources for information, records keeping forms for lab results, daily health questionnaire, etc, combined into a small notebook and given out
229. Some Albertans do not proceed with treatment because of the fear of the unknown or perceived poor outcome, timely data on outcomes need to be published
230. Patients need information on communication strategies pre-op to have success post-op
231. Mentoring videos from long term survivors accessible through internet as the internet is first place you go once diagnosed
232. More cohesive assistance with mental health during treatment. Significant depression, anxiety, fear increase the challenges of treatment, and medication without counseling is not adequate. One on one and group discussions are critical
233. We need more effort towards long term survivors mentoring newly diagnosed patients. Many questions arise that may not be directly treatment related & difficult to answer for new patients. Long term patients often have first hand experience that helps
234. Dental challenges aren't adequately managed. Complete extraction recommended, with limited restoration options. Dentures seldom work well, implants usually needed but most can't afford them. SK has special funding available for this, not Alberta
235. Follow up. Well beyond the 5 year norm, Oral cancer patients frequently develop treatment related problems, i.e. oesteoradionecrosis, graft resorption, fibrosis, baroreflex disorders, etc.. These significantly effect quality of life.
236. Make it clearly understood how important exercise is for the entire body and the cancer affected area, in order to properly rehabilitate. Exercise is not to be done for a just week or a month but to continue forever, to ensure the maximum recovery.
237. Be introduced to what therapies are available re-enable the ability to swallow properly.
238. Be introduced to what therapies are available re-enable the ability to speak clearly.
239. Have interaction with the head and neck cancer support group BEFORE the surgery and treatments, so as to know, from a patient perspective, what to expect and how to deal with things that will come up.
240. Have somebody with you during treatment
241. Eating may become an issue. Try to eat
242. Take advantage of as many information seminars

243. Ask questions
244. Keep your mind active
245. Be aware of changes to yourself
246. Transportation to and from treatment
247. Keep family informed
248. Comfortable accommodations while in treatment
249. Stay as active as possible
250. Keep a positive attitude
